# Traditional Processing of *Arum maculatum* L. and Apparent α-Amylase Inhibition: An Exploratory In Vitro and In Silico Study

**DOI:** 10.3390/molecules31213994

**Published:** 2026-11-05

**Authors:** Zafer Ömer Özdemir, Ezgi Keskin, Mustafa Niğde, Duygu Mısırlı, Ebru Özdemir Nath, Mahfuz Elmastaş

**Affiliations:** 1 Department of Analytical Chemistry, Hamidiye Faculty of Pharmacy, University of Health Sciences Türkiye, 34668 İstanbul, Türkiye; 2 Hamidiye Faculty of Pharmacy, University of Health Sciences Türkiye, 34668 İstanbul, Türkiye; ezgikeskin3807@gmail.com; 3 Department of Bioengineering, Faculty of Chemical and Metallurgical Engineering, Yildiz Technical University, 34220 İstanbul, Türkiye; mnigde@yildiz.edu.tr; 4 Department of Biochemistry, Hamidiye Faculty of Pharmacy, University of Health Sciences Türkiye, 34668 İstanbul, Türkiye; duygu.misirli@sbu.edu.tr; 5 Department of Pharmacognosy, Faculty of Pharmacy, Altınbaş University, Bakırköy Health Campus, 34145 İstanbul, Türkiye; ebru.ozdemir@altinbas.edu.tr

**Keywords:** *Arum maculatum*, α-amylase inhibition, phenolic compounds, traditional processing, decoction, molecular docking

## Abstract

*Arum maculatum* L. is a traditionally consumed but toxic and irritant plant that must be processed before use. This exploratory study examined whether three preparations—an ethanol:water (80:20, *v*/*v*) extract of dried material (E1), a corresponding extract of fresh material (E2) and an aqueous decoction of fresh material (E3) mirroring traditional boiling—differ in apparent α-amylase inhibition, and whether their previously reported phenolic marker profiles (high-performance liquid chromatography with photodiode array detection, HPLC–PDA) account for these differences. Inhibition of *Aspergillus oryzae* α-amylase was measured by the starch–iodine method at a single extract concentration and two enzyme loadings, using one extract per preparation and analytical triplicates. All extracts gave higher apparent inhibition values (75.13–90.80%) than the assay-internal acarbose control (67.28–68.15%) at the same nominal mass concentration (40 µg/mL), with E3 highest at the higher enzyme loading. The previously quantified markers did not account for this pattern. Docking against fungal (3VX0, 7TAA) and human (2QV4, 4GQR) α-amylases placed all markers in the catalytic cleft, a hypothesis-generating result that does not establish binding affinity. These descriptive differences indicate that processing is associated with different apparent α-amylase inhibition, whereas the active constituents, possible assay interference and the safety of the preparations remain to be established.

## 1. Introduction

Type 2 diabetes mellitus (T2DM) is characterized by chronic hyperglycemia and is strongly influenced by postprandial glucose excursions. A key nutritional target for attenuating postprandial hyperglycemia is the inhibition of carbohydrate-hydrolyzing enzymes, particularly pancreatic α-amylase and downstream, intestinal α-glucosidases, thereby slowing starch digestion and glucose absorption [1,2]. Although pharmacological inhibitors such as acarbose are clinically effective, their gastrointestinal adverse effects have motivated continued interest in food- and plant-derived enzyme inhibitors as potential dietary or functional-ingredient strategies. In this context, polyphenols have received substantial attention because many phenolic acids and flavonoids can bind digestive enzymes through hydrogen bonding and hydrophobic/π–π interactions, producing measurable inhibition that depends on structural features (e.g., hydroxylation pattern, glycosylation) and on the food matrix [3,4].

Among wild edible plants, *Arum maculatum* L. (Araceae) is a particularly instructive case because it is traditionally used but intrinsically hazardous when raw. Multiple sources indicate that *A. maculatum* contains needle-like calcium oxalate raphides and other irritant/toxic constituents that can cause intense oral and gastrointestinal irritation and, in severe exposures, airway compromise [5,6,7]. Consistent with this risk profile, ethnobotanical records emphasize that consumption requires processing—drying, boiling, or fermentation followed by prolonged heating—to reduce toxic principles and render the plant acceptable as a seasonal food in certain regions [8,9,10]. Such processing is not chemically neutral: thermal treatment, dehydration, and aqueous extraction can remodel the phenolic composition through leaching, hydrolysis, oxidation, and deglycosylation, and such structure-dependent shifts could, in principle, alter α-amylase inhibitory activity [4].

Although *A. maculatum* has been investigated for bioactivity under different solvent and extraction systems, the literature still lacks ethnobotany-aligned, head-to-head comparisons that connect folk processing analogs (fresh vs. dried preparations and decoction-type products) to (i) HPLC-resolved phenolic marker shifts and (ii) α-amylase inhibition in a single experimental frame [8,11].

A previous study by our group on the same three preparations (E1–E3) reported their antioxidant capacity (DPPH, CUPRAC), with E1 showing the highest activity, together with the HPLC–PDA quantification of five phenolic markers [12]. The marker concentrations in Table 1 and the corresponding calibration parameters (Supplementary Materials, Table S1) were therefore published in [12] as mean values; they are reproduced here, together with the standard deviations of the underlying analytical replicates, because they are the chemical basis against which the new activity data are interpreted. The data reported here for the first time are the α-amylase inhibition measurements (Table 2), the expression of marker contents per gram of fresh material (Supplementary Materials, Table S5), the descriptive comparison of marker contents with inhibition, and the docking and in silico ADME analyses. Building on the previously reported marker profiles, we asked whether processing-related differences in these profiles are sufficient to explain differences in apparent α-amylase inhibition.

Therefore, the present study evaluates *A. maculatum* preparations designed to mirror folk processing logic (fresh and dried plant-derived ethanolic extracts and a decoction-type preparation), compares their apparent α-amylase inhibition in an end-point starch–iodine assay, and interprets this activity against their previously reported targeted high-performance liquid chromatography with photodiode array detection (HPLC–PDA) marker profiles [12]. Molecular docking against fungal and human α-amylase structures is used only as a hypothesis-generating tool to explore possible ligand–enzyme interactions of these markers. Because each preparation was represented by a single extract, the study was designed as an exploratory, descriptive comparison.

## 2. Results

### 2.1. Phenolic Marker Profiles of the Preparations (HPLC–PDA; Previously Reported Data)

The HPLC–PDA marker data summarized in this section were previously reported in [12] and are reproduced here because they are required to interpret the α-amylase results; only the SDs of the analytical replicates and the fresh-weight recalculation (Supplementary Materials, Table S5) are new. Targeted HPLC profiling quantified five phenolic markers (gallic acid, chlorogenic acid, ferulic acid, apigenin-7-*O*-glucoside, and quercetin) across extracts derived from different traditional/processing scenarios. Gallic acid, chlorogenic acid, and quercetin were detected in all extracts, whereas apigenin-7-*O*-glucoside was only present in the dried-material extract (E1), and ferulic acid was detected in the fresh-material ethanolic extract (E2) and decoction (E3). HPLC–PDA chromatograms for E1, E2, and E3, recorded in the same analyses and shown at the quantification wavelengths, are provided in Supplementary Materials (Figure S1). Calibration parameters (R^2^, limit of detection (LOD)/limit of quantification (LOQ)) and retention times, as reported in [12], are provided in Supplementary Materials (Table S1). The concentrations of the quantified phenolic markers in E1, E2, and E3 are summarized in Table 1.

Expressed per gram of extract, E1 showed the highest “sum of quantified markers” among the five analytes, driven primarily by elevated gallic and chlorogenic acids, while E2 was characterized by the highest quercetin and measurable ferulic acid. E3 contained markedly less quercetin per gram of extract. Because E2 and E3 were prepared from identical amounts of the same fresh material, their contents can also be expressed per gram of fresh material (Supplementary Materials, Table S5), on which basis the decoction yielded 3–7-fold more gallic and chlorogenic acid and only ≈24% less quercetin than E2. However, E2 and E3 also differed in solvent composition (ethanol:water 80:20 vs. water) and solvent volume (300 vs. 500 mL; material-to-solvent ratios of about 1:2 and 1:3.4, *w*/*v*), both of which affect extraction efficiency [13]. The higher fresh-weight recovery of phenolic acids in E3 therefore reflects the combined decoction conditions and cannot be attributed to heating alone; matrix softening and release from bound forms during boiling [14] are possible contributors, and the lower quercetin recovery is consistent with the known degradation of quercetin during heating in aqueous media [15]. Because E1 was prepared from dried material and E2/E3 from fresh material of undetermined moisture content, comparisons between E1 and E2/E3 reflect the combined effects of processing type and extraction efficiency rather than a biomass-normalized contrast; only E2 and E3 share a common (fresh-weight) basis, and even this comparison is confounded by the solvent differences noted above. All between-preparation comparisons in this section are descriptive, because each preparation was represented by a single extract (Section 2.6).

### 2.2. Apparent α-Amylase Inhibition: Effect of Processing Scenario

α-Amylase inhibitory activity was evaluated by the starch–iodine method, using acarbose as the reference inhibitor. Because this end-point method reports the residual starch–iodine color rather than enzyme activity directly, and because only a single extract concentration was tested, the values are referred to as apparent inhibition throughout; they do not define potency or an inhibition mechanism. Under the tested conditions, all three extracts showed numerically higher inhibition values than the acarbose control at the same nominal mass concentration (40 µg/mL). This comparison is mass- rather than molarity-based and contrasts a crude multicomponent extract with a single purified drug against a fungal enzyme for which acarbose is not optimized; it should therefore be read as an assay-internal reference, not as evidence of superior pharmacological potency. The inhibition values obtained under the two enzyme-loading conditions are summarized in Table 2.

At 25 µL enzyme loading, the apparent inhibition values of the three extracts lay between 77.50% (E2) and 81.09% (E1), compared with 67.28% for acarbose; at 50 µL enzyme loading, E3 gave the highest value (90.80%), followed by E2, E1 and acarbose (Table 2). Because the reported *n* = 3 values represent analytical replicates from single extract preparations, these differences are presented descriptively rather than as independent inferential comparisons. Two additional points are noteworthy. First, the inhibitory response pattern shifted with enzyme load: at the lower load the three extracts differed by less than 4 percentage points (within about two SDs), whereas at the higher load E3 gave the highest value. Because inhibition by a reversible inhibitor at a fixed concentration would not be expected to increase when the amount of enzyme is doubled, this pattern is not readily explained by simple reversible inhibition. Non-linear end-point behavior of the starch–iodine assay is one possible explanation, but it remains a hypothesis: demonstrating it would require progress curves, substrate- and inhibitor-concentration series and kinetic analysis, none of which were performed, and the present data cannot distinguish it from matrix-specific interference (see below) or other assay effects. Second, published data on the α-amylase inhibitory activity of *A. maculatum* are scarce and heterogeneous. Using the same starch–iodine readout, Aslan et al. could calculate an IC_50_ value only for the ethanolic leaf extract, which reached an inhibitory effect comparable to that of acarbose only when four-fold concentrated, whereas sugar-rich seed and berry extracts gave unstable results [16]. For the congeneric *A. elongatum*, α-amylase inhibition likewise varied markedly with the extraction solvent [17]. Against this background, the comparatively high inhibition observed here suggests that extraction/processing choices and assay conditions (including test concentration, enzyme source and interference controls) can materially affect apparent potency.

It should be noted that the relatively modest inhibition observed for acarbose (approximately 67–68% at the same nominal concentration) compared with the plant extracts is consistent with the well-documented enzyme-source dependence of this inhibitor: acarbose is a potent inhibitor of mammalian α-amylases, but its affinity for the *A. oryzae* enzyme is markedly lower [18]. In a comparative kinetic study, the inhibition constant (K_i_) of acarbose was 270 µM for *A. oryzae* α-amylase, compared with 1.27 µM for human salivary and 0.80 µM for porcine pancreatic α-amylase, corresponding to approximately 213-fold and 338-fold higher K_i_ values for the fungal enzyme, respectively [18]. Despite their structural homology, fungal and mammalian α-amylases also differ markedly in functional behavior, so results obtained with the *A. oryzae* enzyme cannot be directly extrapolated to the human enzyme [19]. For a purely non-competitive inhibitor, a K_i_ of 270 µM would correspond to about 56% inhibition at the ≈344–354 µM acarbose present during incubation, and a competitive inhibitor would give less (acarbose showed mixed, non-competitive kinetics with this enzyme [18]); the somewhat higher values observed here may reflect the non-linear relationship between residual-starch end-point readouts and initial-rate inhibition. The acarbose values should therefore be interpreted as an assay-internal positive control rather than as a benchmark of expected pharmacological potency. Future studies employing porcine pancreatic α-amylase would yield a more clinically translatable reference comparison.

The starch–iodine readout is also susceptible to matrix-specific interference by plant extracts [20,21]. Redox-active/antioxidant constituents can consume iodine and weaken color development, which would lead to underestimation of inhibition [20]; conversely, constituents that interact with starch or with the enzyme (e.g., polyphenol–starch or polyphenol–protein complexes) and co-extracted carbohydrates that compete with starch as substrate could shift the residual starch–iodine signal in either direction. The inhibitor blank used here (extract in buffer, without substrate or enzyme) corrects for the intrinsic absorbance of the extracts but not for these reaction-dependent effects. The net direction and magnitude of matrix interference therefore cannot be established from the present data, and the 75–91% values should not be interpreted as an effect on the catalytic activity of α-amylase alone until they are confirmed by an orthogonal method (e.g., a reducing-sugar or chromogenic-substrate assay with appropriate matrix controls).

The smaller separation among extracts at the lower enzyme loading may reflect the higher nominal extract-to-enzyme ratio and the limited discriminatory range of the assay under these conditions. At the higher enzyme loading, differences among preparations became more apparent, although they remain descriptive. Because the identities and molar concentrations of the active constituents in the crude extracts are not fully defined, no mechanistic interpretation is possible. Substrate-dependent kinetic studies and K_i_ determinations are required to establish the inhibition mechanism.

### 2.3. Descriptive Comparison of Phenolic Markers and Apparent α-Amylase Inhibition

**Figure 1:**
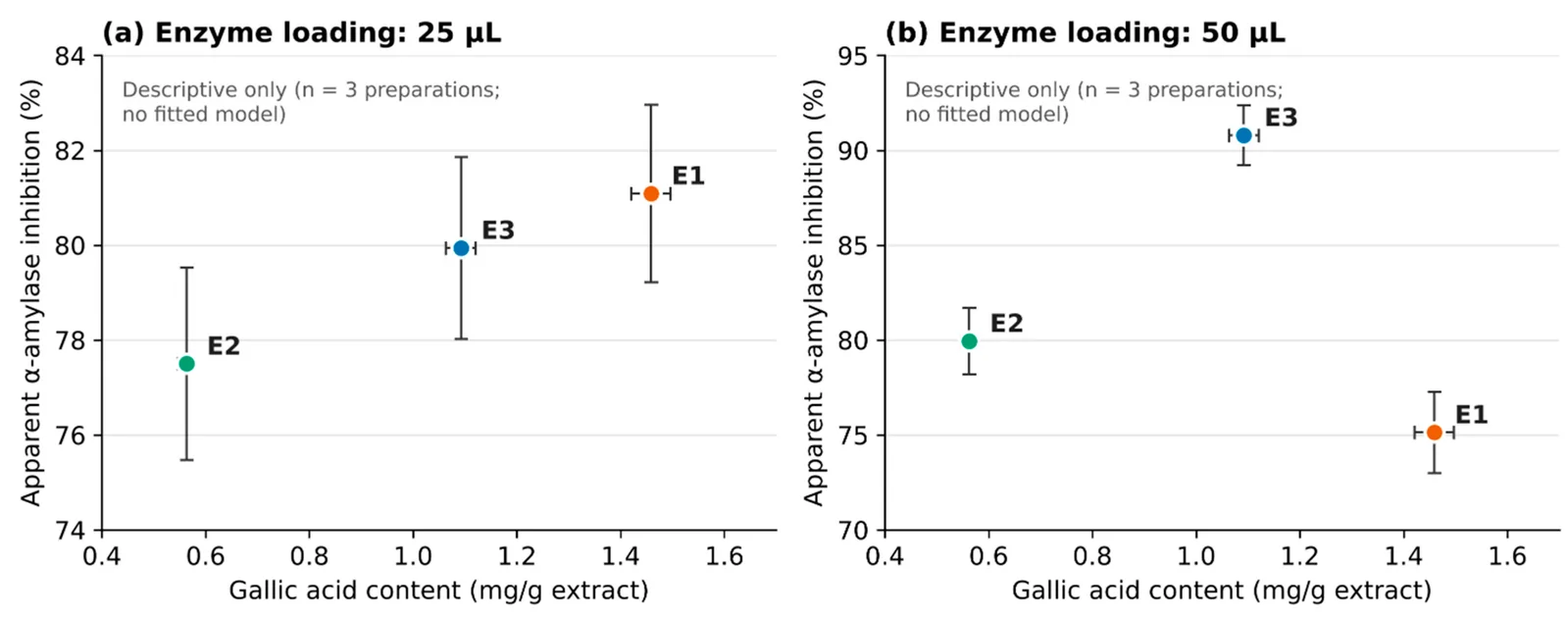
Gallic acid content (previously reported in [12]) plotted against apparent α-amylase inhibition for the three preparations E1, E2, and E3 at (**a**) 25 µL and (**b**) 50 µL enzyme loading. Points are means; error bars are SDs of three analytical replicates of a single extract per preparation. Because only three preparations were available (df = 1), no regression line, R^2^ or *p*-value is shown, and the plots are descriptive only.

To explore descriptively whether the differences in apparent α-amylase inhibition paralleled the content of a single marker, gallic acid content (the marker that varied most among the preparations) was plotted against inhibition at each enzyme loading (Figure 1). Because each enzyme loading provides only three data points (one per preparation; df = 1 for a linear fit), no regression model, coefficient of determination or *p*-value is reported, and the plots are not used for inferential purposes. At 25 µL enzyme loading, the rank order of inhibition (E1 > E3 > E2) matched that of gallic acid content, although the three inhibition values differed by less than 4 percentage points (within about two analytical SDs); at 50 µL enzyme loading, it did not (E3 > E2 > E1). With only three preparations, neither observation can be taken as evidence for or against an association between gallic acid and inhibition; they show only that this marker does not provide a consistent description of the inhibition pattern under both assay conditions.

A notable observation concerns E3, which exhibited the strongest inhibition at 50 µL enzyme loading (90.80 ± 1.58%) despite containing the lowest quercetin level and a lower sum of quantified markers than E1. This dissociation between the targeted marker profile and the inhibitory response is compatible with several non-mutually-exclusive hypotheses, none of which was tested here: (i) phenolic or non-phenolic constituents outside the 14-analyte targeted panel; (ii) processing-derived transformation products generated during the decoction step (e.g., partial hydrolysis or thermal rearrangements); (iii) non-additive mixture effects (additivity, synergy, or antagonism) among the co-extracted compounds; and (iv) matrix-specific interference with the starch–iodine readout (Section 2.2).

Regarding hypothesis (ii), thermal processing during decoction (E3) could generate transformation products that retain or even enhance α-amylase inhibitory activity while no longer being detected by the targeted HPLC method. The available marker data do not resolve this question: apigenin-7-*O*-glucoside was not detected in the fresh-material extract (E2), the corresponding aglycone was not part of the targeted panel, and caffeic acid, a hydrolysis product of chlorogenic acid, was not detected in E3. Decoction-derived products therefore cannot be identified from the present data, and untargeted LC–MS/MS profiling of E2 and E3 would be required.

### 2.4. Molecular Docking Results

**Figure 2:**
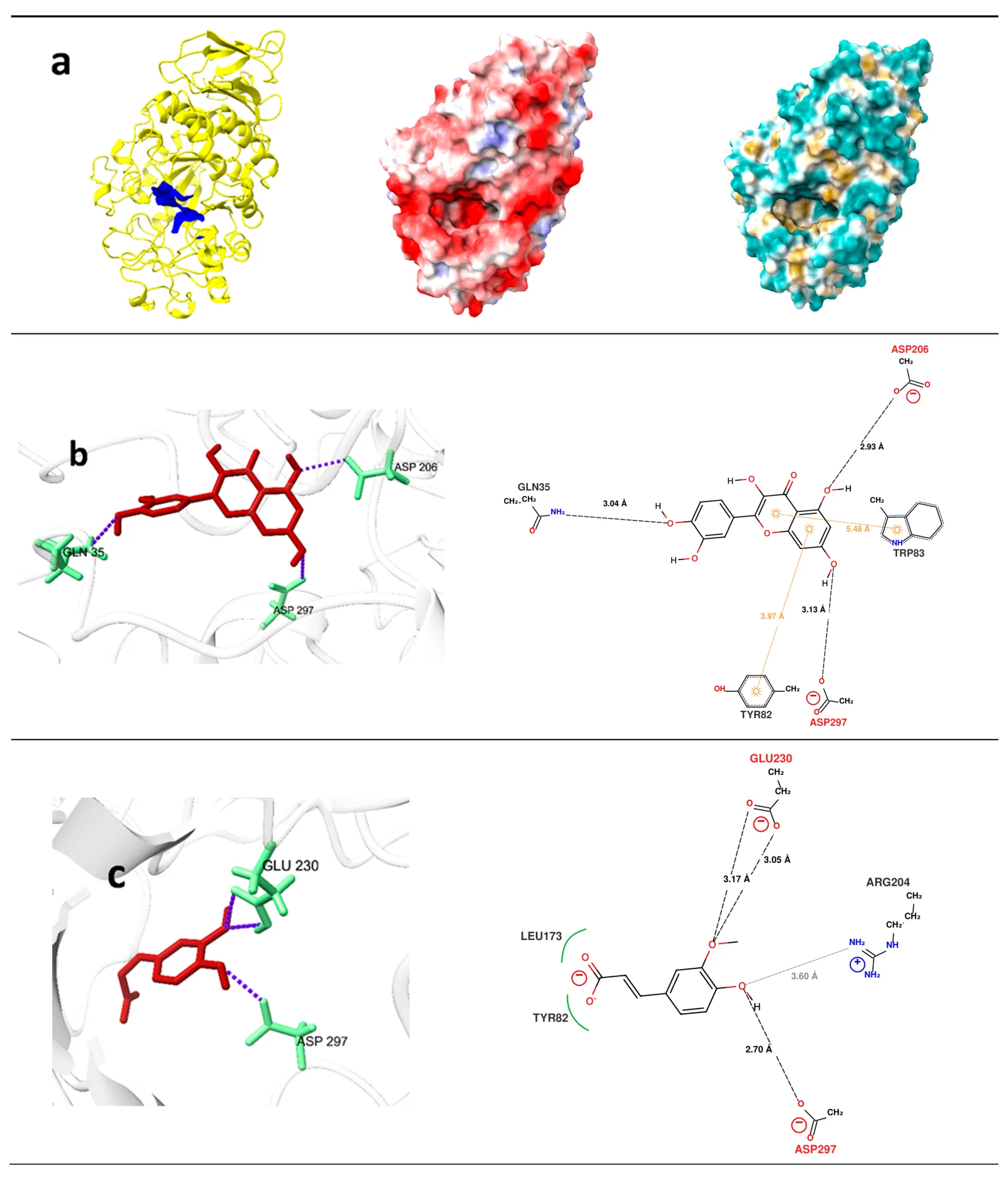

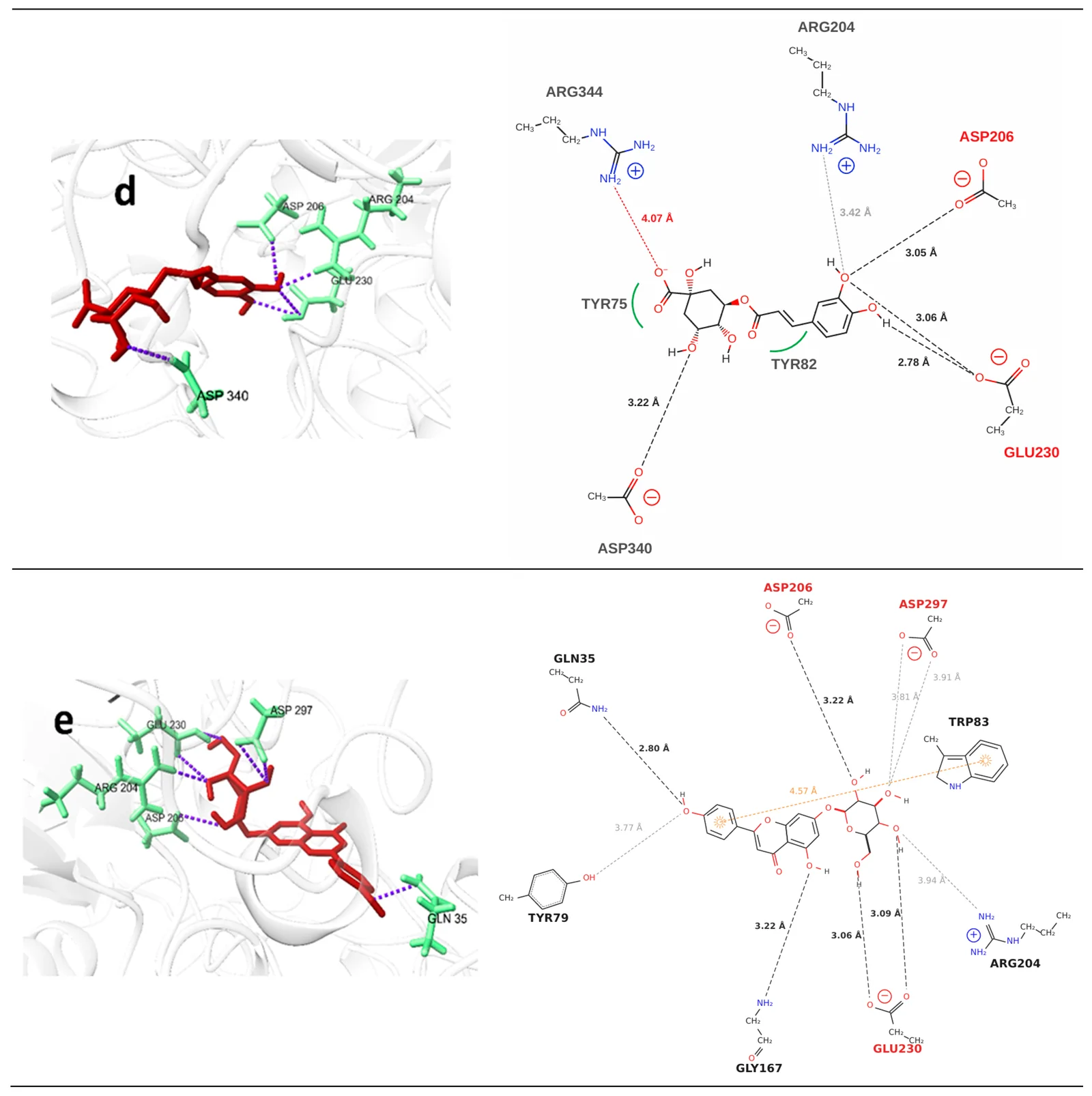

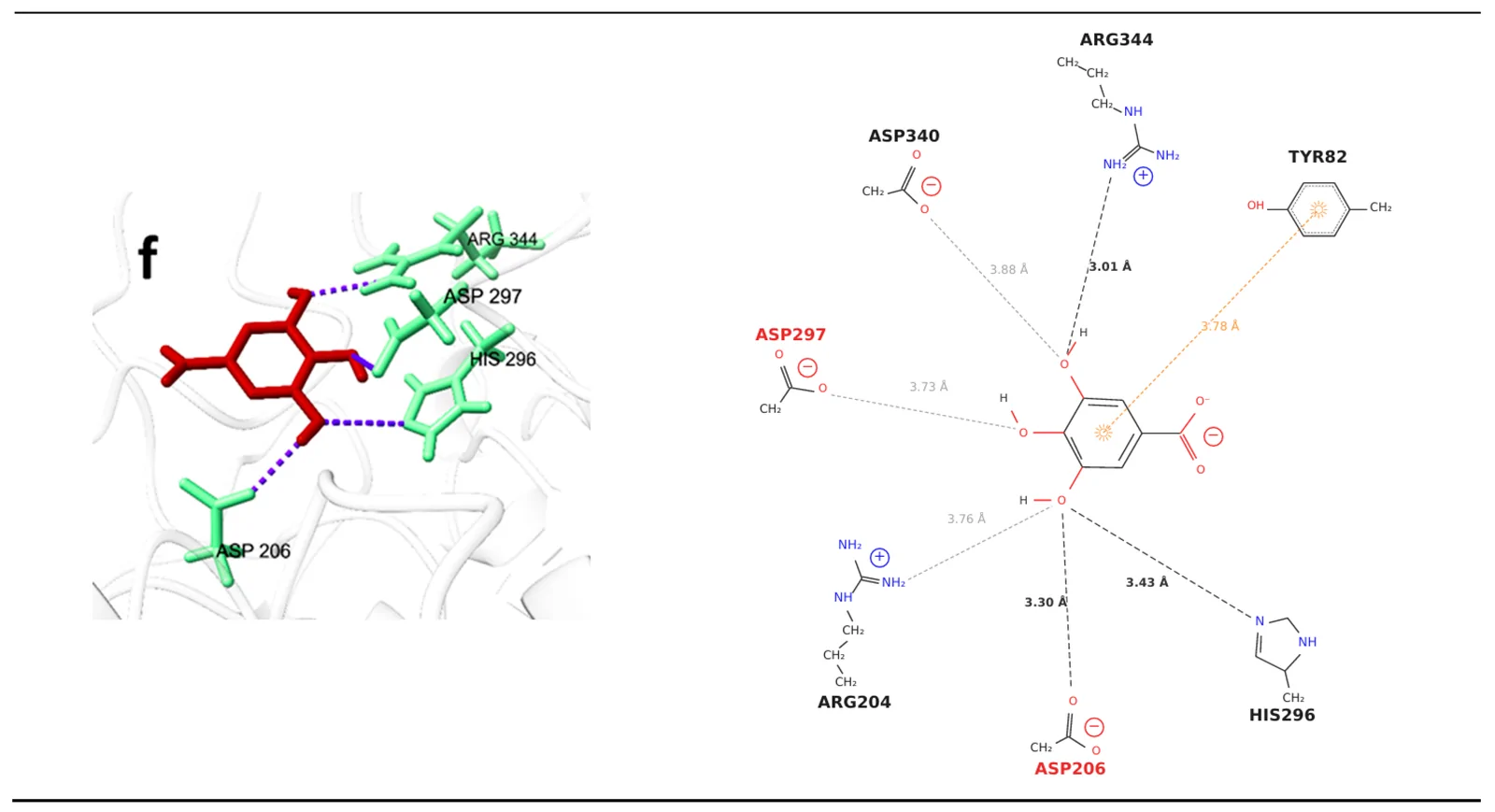
Three-dimensional and two-dimensional visualizations of the top complexes for 7TAA, with the crystallographic structure selected as the primary representative of the experimental system. From top to bottom: (**a**) general view of the enzyme with its active site indicated, along with electrostatic and hydrophobic states, (**b**) quercetin, (**c**) ferulic acid, (**d**) chlorogenic acid, (**e**) apigenin-7-*O*-glucoside, (**f**) gallic acid. The 3D view is shown only with the most important interacting residues for clarity. Symbols used in the 2D interaction diagrams: hydrogen bonds (dashed black lines), possible polar contacts (dashed gray lines), hydrophobic contacts (green arcs), π–π stacking (dashed orange lines with a terminal circle), and salt bridges (dashed red lines).

Although the experimental inhibition assay was based on fungal α-amylase, docking was performed against four α-amylase structures spanning both fungal (3VX0, 7TAA) and human (2QV4, 4GQR) origins as a hypothesis-generating exercise to explore whether the binding-site recognition of the phenolic markers was conserved across these enzyme classes. Redocking reproduced the crystallographic ligand poses of the three holo structures (7TAA, 2QV4, 4GQR) with symmetry-corrected root-mean-square deviation (RMSD) values ≤ 1.16 Å and SuCOS scores ≥ 0.88 only when active-site crystallographic waters were retained; with fully desolvated receptors, which were used for phytochemical docking, the corresponding RMSD values were 6.78, 7.75 and 2.34 Å (Supplementary Materials, Table S3). The phytochemical poses reported below should therefore be regarded as structural hypotheses rather than validated binding modes; the apo structure 3VX0 was excluded from redock validation as no co-crystal ligand was available. The receptor–ligand combinations are summarized in Figure 2 (representative complexes for 7TAA) and in Supplementary Materials (Figures S2–S4 for 3VX0, 2QV4, and 4GQR).

The diagrams for the 7TAA poses (Figure 2) indicate a recurrent interaction region organized around the catalytic acidic environment. For four of the five phytochemicals, Glu230 and/or Asp297 emerge as the most consistent catalytic contacts (gallic acid forms only borderline contacts with these residues), Arg204 repeatedly appears as a secondary polar partner, and aromatic contacts are ligand-dependent. H-bonds reported by PLIP with donor–acceptor distances longer than 3.5 Å or donor–H–acceptor angles smaller than 110° were considered weak/borderline polar contacts. For chlorogenic acid, H-bonds were observed to Glu230 (2.78–3.06 Å, 124–138°), Asp340 (3.22 Å, 165°), Arg204 (3.42 Å, 167°), and Arg344 (3.06 Å, 103°), together with a 4.07 Å salt bridge to Arg344. The pose also displayed hydrophobic contacts with Tyr75/Tyr82 and a nearby Asp206. Quercetin is comparatively compact, retaining H-bonds to Asp206 (2.93 Å, 138°), Asp297 (3.13 Å, 126°), and Gln35 (3.04 Å, 133°), while Tyr82 and Trp83 are located against the aromatic region of the ligand. Gallic acid displays the densest polar network, although several contacts are relatively long, with polar bonds to His296 (3.43 Å, 138°), Asp297 (3.73 Å, 143°), Asp340 (3.96 Å, 105°), Arg204 (3.75–4.08 Å, 139–151°), and Arg344 (3.01–3.83 Å, 129–160°); the 2D view further highlights Asp206 and Tyr82 around this pose. Ferulic acid shows a simpler pattern centered on Glu230/Asp297/Arg204, with polar contacts of 3.17 Å, 163° (Glu230), 4.08 Å, 130° (Asp297), and 3.60 Å, 171° (Arg204), accompanied by Tyr82 and Leu173 hydrophobic interactions. Apigenin-7-*O*-glucoside spans the broadest footprint in the 7TAA set, with polar bonds to Tyr79 (3.77 Å, 109°), Glu230 (3.09 Å, 165°), Asp297 (3.81 Å, 119°), Gln35 (2.80 Å, 114°), Gly167 (3.22 Å, 132°), and Arg204 (3.84 Å, 161°). Notably, this network also extends toward Asp206 and Trp83.

As a complementary tool, molecular docking identified plausible binding modes of the quantified phytochemicals within the catalytic pocket of α-amylase; given their low concentrations in the assay, these poses illustrate possible interactions rather than explain the inhibition measured for the extracts. Relative to the redocked co-crystal ligands, whose scores serve only as an approximate benchmark (Table 3), phytochemical Vina scores were less favorable for 7TAA and 2QV4. For 4GQR, however, quercetin (−9.53 kcal/mol) showed a Vina score similar to and slightly more negative than that of the redocked co-crystal ligand myricetin (−9.45 kcal/mol). These score comparisons should be interpreted descriptively and not as direct evidence of experimental binding affinity. All studied phytochemicals nevertheless showed interactions with catalytic residues across the tested fungal and human α-amylase structures. Docking scores and poses do not constitute evidence of experimental affinity, K_i_, potency or inhibition mechanism, and the poses obtained for the human enzymes are an in silico extrapolation only, because inhibition was measured exclusively with the fungal enzyme. A comparative heat map of AutoDock Vina binding scores across the tested receptor–ligand combinations is provided in Supplementary Materials (Figure S6). Pose selection was based not only on docking scores but also on interaction quality, geometric validity and quality checks; additional pose-selection metrics are provided in Supplementary Materials (Table S4), and representative interaction visualizations are shown in Supplementary Materials (Figures S2–S4).

In comparison to the experimental inhibition results, apigenin-7-*O*-glucoside produced the strongest absolute Vina scores in two of the four α-amylase structures tested (7TAA: −10.14; 2QV4: −10.66 kcal/mol), which partly reflects the known tendency of the Vina scoring function to favor larger ligands; the CNN pose scores of these poses (0.14 for 7TAA and 0.49 for 2QV4) were lower than those of quercetin (0.26 and 0.71; Table 3), indicating lower confidence in the apigenin-7-*O*-glucoside poses. In the remaining two structures, quercetin had the most negative Vina score (3VX0: −9.27; 4GQR: −9.53 kcal/mol). Quercetin ranked first or second by Vina score in all four receptors (−9.27 to −10.36 kcal/mol). Chlorogenic acid showed intermediate scores (−7.89 to −9.17 kcal/mol), and gallic acid and ferulic acid were generally weaker by score, consistent with their smaller molecular volume and fewer hydrogen-bond donor/acceptor atoms. Notably, E3 (the decoction) exhibited the highest experimental inhibition at the 50 µL enzyme load despite containing the lowest quercetin level and no detectable apigenin-7-*O*-glucoside, indicating that docking of a few markers cannot fully predict complex mixture activity. It is possible that thermal processing transforms some of the original phytochemicals into derivatives (e.g., aglycones, oxidation products, polymerized polyphenols) that retain or even enhance α-amylase interaction while remaining outside the targeted HPLC panel, but this hypothesis could not be tested with the present data.

### 2.5. Practical Implications

Taken together, the three preparations, which differed in processing and extraction conditions, also differed in their previously reported phenolic marker composition and in apparent α-amylase inhibition in an end-point assay with a fungal enzyme. A comparative radar plot summarizing the normalized phenolic marker and α-amylase inhibition profiles of the three processing scenarios is provided in Supplementary Materials (Figure S7). These are descriptive in vitro observations on single preparations. They are evidence of biological activity under assay conditions only; they provide no evidence of an effect on postprandial glycemia in vivo and no evidence that any of the preparations are safe to consume (see Section 3).

### 2.6. Limitations

Docking results are predictive and require experimental validation. Because inhibition assays were not performed for the human α-amylase homologs, these docking results should not be interpreted as direct evidence of stronger or weaker inhibition relative to the fungal enzyme system. Moreover, the limited conformational sampling used here may not capture induced-fit effects; broader ensemble docking and molecular dynamics simulations could therefore strengthen future analyses. Accordingly, all docking results, including those for the human enzymes, are used here exclusively as hypothesis-generating.

Phenolic compounds can act as electron donors (electron shuttling), potentially reducing I_2_ to I^−^ and interfering with the starch–iodine complex formation. Because such reduction lowers the residual starch–iodine absorbance, this redox-mediated interference would, if uncorrected, lead to underestimation of α-amylase inhibition in extracts rich in antioxidant phenolics [20]. Other matrix effects could act in the opposite direction (Section 2.2). The inhibitor blank corrects for the intrinsic absorbance of the extracts but not for these reaction-dependent effects, so the net bias of the reported values is unknown; this must be addressed with an orthogonal readout (e.g., a DNS-based or chromogenic-substrate assay). Furthermore, the assay was conducted at 37 °C, below the temperature optimum (~50 °C) and upper thermal-stability range (~55 °C) reported for *A. oryzae* α-amylase [22,23], to keep the incubation conditions comparable with commonly used screening protocols [19,24]; because the enzyme is of fungal origin, this choice does not by itself confer physiological relevance, and future studies could repeat the assay at the enzyme’s optimum and with porcine or human pancreatic α-amylase.

An important design consideration is that the three processing scenarios were conceived as practical analogs of the dried, fresh and boiled states of the plant rather than biomass-controlled comparisons; accordingly, E1 started from dried material (5.33 g) whereas E2 and E3 used fresh tissue (146.85 g each), and the moisture content of the fresh material was not determined. Because the water contained in the fresh tissue diluted the extraction solvent, the effective ethanol content during E2 extraction was lower than the nominal 80% (*v*/*v*), and the solid-to-solvent ratio also differed markedly from that of E1; E1–E2 differences therefore cannot be attributed to drying alone. Likewise, E2 and E3 differed in solvent composition and volume (300 mL ethanol:water vs. 500 mL water), so the E2–E3 contrast reflects the combined preparation conditions rather than heating alone. In addition, neither liquid nitrogen nor dry ice was used during comminution of the plant material, so enzymatic or oxidative changes in labile phenolics during grinding cannot be excluded. Future studies should include dry-matter determination of fresh material to enable yield and phenolic normalization on a common dry-weight basis. Additionally, this study is limited by the use of an in vitro enzyme assay at a single test concentration; therefore, future work should include dose–response curves and IC_50_ values and, ideally, inhibition kinetics to clarify the mode of inhibition. Because starch–iodine assays may be affected by redox-active constituents and sample color, rigorous blank correction is essential and an orthogonal method (e.g., DNS-based reducing sugar readout or a chromogenic substrate assay) should be used to confirm the magnitude of inhibition. Phenolic profiling was targeted to selected markers; untargeted LC–MS (or expanded standards) would better capture processing-derived transformation products and mixture effects. Finally, because *A. maculatum* is recognized as potentially irritant/toxic when raw, translating these findings to food relevance requires explicit safety-oriented characterization of processing outcomes (e.g., irritant/toxic principles as appropriate) and, if the scope allows, simulated gastrointestinal digestion to evaluate stability and bioaccessibility of inhibitory constituents.

A further methodological limitation is that each processing condition was represented by a single extraction batch, with triplicate analytical measurements used to characterize measurement precision. This design does not estimate between-extraction variability and therefore limits generalization of differences among E1, E2, and E3 beyond the preparations analyzed here. Future studies should include at least three independently prepared extraction batches per processing condition, together with full dose–response designs and appropriate inferential statistics based on those independent experimental units. Consequently, all differences among E1, E2 and E3 reported in this study are descriptive: they characterize the specific preparations analyzed and should not be generalized to *A. maculatum* material or to the processing methods as such.

Because inhibition was evaluated at a single substrate concentration (≈1.7 mg/mL starch during the enzymatic incubation), the present design does not permit any classification of the inhibition mechanism (competitive, non-competitive, or mixed); throughout this work, “inhibition” denotes a reduction in the end-point starch-hydrolysis signal under the assay conditions (apparent inhibition), not a characterized enzyme–inhibitor interaction. Future work should include substrate-concentration-dependent assays to derive K_m_ and K_i_ values.

## 3. Discussion

The three preparation scenarios of *Arum maculatum* differed in their previously reported phenolic marker composition [12], and all three preparations showed numerically higher apparent α-amylase inhibition values than acarbose under the tested assay conditions at the same nominal mass concentration. The inhibitory pattern, however, did not follow the marker profile: the decoction (E3), which had the lowest quercetin content and the lowest sum of quantified markers per gram of extract, gave the highest inhibition at the higher enzyme loading. Because the five quantified markers together accounted for only about 0.2–0.3% (*w*/*w*) of each extract and were present at no more than about 2 µM during the enzymatic incubation, they are unlikely to explain the observed inhibition on their own. Docking suggests that these compounds can occupy the catalytic cleft, but the apparent activity of the extracts is more plausibly attributable to non-targeted constituents, mixture effects or assay-matrix effects. Within the limits of this exploratory, single-preparation design, the processing scenarios were therefore associated with differences in both the phenolic profile and the apparent α-amylase inhibition of *A. maculatum*, through constituents that remain to be identified.

The marker differences between preparations are consistent with the two opposing effects generally described for thermal and hydrothermal processing of plant foods: degradation and leaching of labile compounds on the one hand, and matrix softening that increases extractability on the other, with the net outcome depending on the compound, the matrix and the processing parameters [14]. The low quercetin content of the decoction agrees with the marked degradation of quercetin reported during heating in aqueous model systems, in which cleavage products such as protocatechuic acid were formed [15]. Comparison with other *A. maculatum* studies is limited, because the published data were obtained with different plant parts, solvents and extraction techniques, which themselves strongly modify phenolic recovery [11,13,16]; for example, the phenolic content and antioxidant activity of *A. maculatum* leaf extracts have been shown to depend on both the solvent and the extraction method [11]. The present comparison adds the processing dimension, but because solvent composition, solvent volume and the physical state of the material varied together, it cannot isolate the effect of drying or boiling as such.

A central question raised by these data is whether the quantified markers could plausibly contribute to the measured inhibition. During the enzymatic incubation, each marker was present at no more than about 2 µM. For comparison, acarbose, a potent inhibitor of mammalian α-amylases, has a K_i_ of about 270 µM for the *A. oryzae* enzyme [18] and gave only 67–68% apparent inhibition at about 350 µM in the present assay, and in a comparative study even the most effective flavonoid inhibitors of porcine pancreatic α-amylase (luteolin, myricetin and quercetin) were distinguished by IC_50_ values below 500 µM [3]. For the markers to account for 75–91% apparent inhibition at ≤2 µM, they would have to be more than two orders of magnitude more potent against the fungal enzyme than acarbose, which is implausible. This quantitative argument, rather than the docking scores, is the main reason why we consider the quantified markers unlikely to be the principal contributors to the measured activity.

Several classes of non-targeted constituents could account for the activity, in line with the strong dependence of α-amylase inhibition on extract type reported for *A. maculatum* and the congeneric *A. elongatum* [16,17]. Tannins and other polymeric polyphenols interact with proteins through their numerous hydroxyl groups and inhibit α-amylase, larger and more complex tannins being the more effective inhibitors [25]; such compounds are poorly represented by a panel of low-molecular-weight standards, and the comparatively high gallic acid contents of E1 and E3 would be consistent with, but do not demonstrate, the presence of hydrolysable gallotannins. Non-phenolic constituents, such as co-extracted carbohydrates or proteins, may also contribute, either through genuine effects on the enzyme or through the assay-matrix effects discussed in Section 2.2. The decoction, which gave the highest apparent inhibition at the higher enzyme loading, also had by far the highest extract yield (5.65 g vs. 1.52 g from the same amount of fresh material), indicating a larger proportion of water-soluble non-phenolic material; this dilutes the markers on a per-gram-of-extract basis and could contribute either to activity or to interference. Distinguishing among these possibilities will require bioassay-guided fractionation, untargeted LC–MS/MS profiling and an orthogonal α-amylase assay.

The safety of *A. maculatum* requires particular emphasis. The plant contains needle-shaped calcium oxalate raphides and other irritant constituents, and poisonings after ingestion of insufficiently processed material have been reported [5,6,7,8]. Drying and boiling are traditionally used to reduce this toxicity [8,9,10], but the extent to which these treatments achieve it has not been quantified for *A. maculatum* and was not assessed in the present study. Evidence from other oxalate-containing foods indicates that soaking and boiling reduce soluble oxalate mainly by leaching into the water, whereas insoluble calcium oxalate—the material constituting raphides—is not removed in this way [26,27]; in taro leaves, for instance, boiling removed about one-third of the soluble oxalate [27]. Moreover, the harmful effects of raphides have been shown to depend largely on synergy with co-occurring proteins such as cysteine proteases, which the needle-shaped crystals help to carry through tissue barriers (the “needle effect”) [28]. Thermal denaturation of such proteins during boiling could plausibly reduce irritancy even when the crystals persist, but this has not been demonstrated for *A. maculatum*, and whether drying alone reduces irritancy is likewise unknown. In the present study, no oxalate, raphide, irritancy or toxicity measurements were performed, and the extracts were filtered and lyophilized, so their composition does not necessarily reflect that of the dried or cooked plant material as consumed. The present data therefore provide evidence of in vitro biological activity only; they do not provide any evidence that the preparations, or *A. maculatum* processed in these ways, are safe to eat.

## 4. Materials and Methods

### 4.1. Plant Material, Authentication, and Processing Scenarios

Plant material (*Arum maculatum* L., Araceae), consisting of leaves and stems, was collected from Kahyalı neighborhood, Tekkeköy district, Samsun, Türkiye (41.2133° N, 36.4631° E; 240 m a.s.l.), in December 2024. Botanical identification was performed by Assoc. Prof. Ebru Özdemir Nath. A voucher specimen (No. 1684) was deposited in the Herbarium of Altınbaş University, Faculty of Pharmacy (HERA). Fresh material was used for E2 and E3 and was comminuted with a blender (8011S, Waring Commercial, Stamford, CT, USA) before extraction; for the “dried” scenario (E1), leaves and stems were air-dried at 25 °C and milled in a laboratory mill (GM 200, Retsch GmbH, Haan, Germany) prior to extraction. Grinding was performed at ambient temperature, without liquid nitrogen or dry ice.

Three processing scenarios were evaluated, designed as practical analogs of the dried, fresh and boiled states in which the plant is handled, rather than as a biomass-normalized comparison: (i) dried plant–ethanol:water extract (E1), representing the traditional dried-herb preparation; (ii) fresh plant–ethanol:water extract (E2), a laboratory reference extract of the unprocessed fresh material (not a food preparation, given the irritant nature of the raw plant); and (iii) decoction extract (E3), representing the traditional aqueous boiling preparation. Starting material amounts therefore reflect practical preparation logic (5.33 g dried powder for E1; 146.85 g fresh material for E2 and E3) rather than equivalent dry-weight inputs. Neither the dry-matter inputs nor the material-to-solvent ratios were matched (Section 4.2 and Section 4.3); these differences were not corrected for, and they were taken into account by restricting all between-preparation comparisons to descriptive statements expressed per gram of extract (and, for E2 and E3 only, per gram of fresh material).

### 4.2. Preparation of Ethanol–Water Extracts (E1, E2)

For E1, dried plant powder was weighed (5.33 g) using an analytical balance (Entris II, Sartorius Lab Instruments GmbH & Co. KG, Göttingen, Germany), transferred into a flask, and extracted with ethanol:water (160 mL:40 mL, *v*/*v*) (material-to-solvent ratio ≈1:37.5, *w*/*v*) under orbital shaking using a shaker (Unimax 1010, Heidolph Instruments GmbH & Co. KG, Schwabach, Germany) for approximately 2 h, followed by filtration. Ethanol was removed using a rotary evaporator (Hei-VAP, Heidolph Instruments GmbH & Co. KG, Schwabach, Germany) at 48 °C. The remaining aqueous phase was stored at −20 °C in a laboratory freezer (MDF-U5412, PHC Corporation, Tokyo, Japan), frozen, and subsequently lyophilized using a freeze dryer (HyperCOOL HC3055, GYROZEN Co., Ltd., Gimpo, South Korea) for approximately 2 weeks.

For E2, fresh leaves and stems (146.85 g) were ground and extracted with ethanol:water (240 mL:60 mL, *v*/*v*) (≈1:2, *w*/*v*, fresh weight) using the same shaking–filtration–evaporation–freezing–lyophilization workflow.

### 4.3. Preparation of Decoction Extract (E3)

For E3, fresh plant material (146.85 g) was boiled with 500 mL distilled water (≈1:3.4, *w*/*v*, fresh weight) for 30 min, filtered, frozen at −20 °C, and lyophilized for approximately 2 weeks to obtain the decoction extract.

Lyophilized extract yields were 1.442 g (E1, from 5.33 g dry material; 27.1% *w*/*w*), 1.52 g (E2, from 146.85 g fresh material), and 5.65 g (E3, from 146.85 g fresh material). Because E2 and E3 were prepared from fresh tissue whose exact moisture content was not determined, a direct yield comparison on a dry-weight basis between E1 and E2/E3 is not possible; all subsequent phenolic and activity data are therefore expressed per gram of lyophilized extract rather than per gram of starting plant material. Extracts were stored at +4 °C until analysis.

### 4.4. Preparation of Working Solutions

Ethanol, acetonitrile, formic acid, potassium dihydrogen phosphate, iodine, potassium iodide, and hydrochloric acid were purchased from Merck KGaA (Darmstadt, Germany). All phenolic reference standards were obtained from Fluka (Honeywell, Seelze, Germany), unless otherwise stated.

For the α-amylase inhibition assay, 10 mg of E1 and E2 were dissolved in 3 mL ethanol and 2 mL distilled water using a vortex mixer (MX-S; DLAB Scientific Co., Ltd., Beijing, China) followed by an ultrasonic bath (SONOREX; BANDELIN electronic GmbH & Co. KG, Berlin, Germany) to obtain 2 mg/mL working solutions. E3 was dissolved in 5 mL distilled water.

Acarbose (A8980, ≥95% by HPLC; Sigma-Aldrich, St. Louis, MO, USA) served as the reference inhibitor and was dissolved in distilled water at 2 mg/mL, matching the working concentration of E1–E3. Solvent-only controls (ethanol:water and water blanks) confirmed that neither vehicle affected α-amylase activity at the volumes used (100 µL per 875–900 µL incubation volume; ≈7% (*v*/*v*) ethanol during the enzymatic incubation).

Considering the assay design (100 µL of 2 mg/mL working solution added to a final read-out volume of 5.0 mL), the concentration of each extract and of acarbose during the enzymatic incubation (875 µL and 900 µL for the 25 and 50 µL enzyme loadings, respectively) was approximately 229 and 222 µg/mL (≈354 and ≈344 µM acarbose; molar mass 645.6 g/mol), corresponding to 40 µg/mL after dilution to the 5.0 mL read-out volume. All samples and the reference inhibitor were therefore evaluated at the same nominal mass-based concentration to allow direct comparison.

For HPLC–PDA analysis, stock solutions were prepared at 1000 µg/mL in the initial mobile phase and vortexed; 2 mL of each sample solution was transferred into vials.

### 4.5. HPLC–PDA Phenolic Profiling

Phenolic compounds were analyzed using an HPLC system (Shimadzu Nexera-i LC-2040C 3D Plus, Shimadzu Corp., Kyoto, Japan) equipped with a PDA detector. Separation was performed on an InertSustain Phenylhexyl column (150 × 4.6 mm, 3 µm; GL Sciences Inc., Tokyo, Japan). Mobile phase A consisted of ultrapure water containing 0.1% formic acid, and mobile phase B consisted of acetonitrile. The gradient program (% B) was: 0.01 min, 5%; 7 min, 9.5%; 20 min, 17%; 35 min, 40%; the total run time was 40.01 min, at the end of which the mobile phase was switched to 100% A and the run was terminated. Before each injection, the column was re-equilibrated at the initial composition (5% B) for 10 min. The flow rate was 1.0 mL/min, the injection volume was 10 µL and the column temperature was maintained at 30 °C. Quantification wavelengths were 271 nm (gallic acid), 325 nm (chlorogenic acid), 322 nm (ferulic acid), 336 nm (apigenin-7-*O*-glucoside) and 254 nm (quercetin) (Supplementary Materials, Table S1). These HPLC–PDA analyses form part of the dataset previously reported in [12].

Calibration was performed using authenticated external standards of gallic acid, epicatechin, cinnamic acid, 4-hydroxybenzoic acid, rutin, quercetin, chlorogenic acid, caffeic acid, ferulic acid, chicoric acid, *p*-coumaric acid, apigenin-7-*O*-glucoside, salicylic acid and naringenin. Analyte identity was confirmed by matching both the retention time and the PDA UV spectrum of each peak with those of the corresponding authenticated reference standard analyzed under identical conditions. Of these 14 standards, five (gallic acid, chlorogenic acid, ferulic acid, apigenin-7-*O*-glucoside and quercetin) gave peaks matching the corresponding reference standard in retention time and UV spectrum in at least one extract and were quantified; the remaining nine were not detected in any of the three preparations.

### 4.6. α-Amylase Inhibition Assay (Starch–Iodine Method)

α-Amylase inhibitory activity was evaluated using the starch–iodine method [29], in which residual (unhydrolyzed) starch reacts with Lugol’s reagent to form a blue–purple complex whose absorbance at 630 nm is inversely related to enzyme activity. A decrease in color intensity relative to the substrate control therefore reflects greater starch hydrolysis, whereas higher absorbance in the presence of a test sample indicates inhibition of the enzyme.

The assay was performed in 50 mM potassium phosphate buffer (pH 7.0 ± 0.05), with the pH measured at 25 °C using a calibrated pH meter (SevenCompact, Mettler-Toledo GmbH, Greifensee, Switzerland). This near-neutral pH corresponds to the intestinal-phase pH (7.0) of the harmonized INFOGEST static in vitro digestion protocol [24] and approximates small-intestinal conditions, where luminal pH rises from mildly acidic values in the duodenum (approximately 4–6) to 7–8 in the ileum [30,31], and where pancreatic α-amylase is the main enzyme responsible for starch hydrolysis in vivo [31]. The incubation temperature was set at 37 °C, as in the INFOGEST protocol [24] and in previous comparative studies using *A. oryzae* α-amylase as a model enzyme [19], rather than at the temperature optimum of the fungal enzyme (~50 °C) [22,23]; this keeps the incubation conditions comparable with commonly used screening protocols. All buffers and reagents were prepared with deionized water and used on the day of analysis.

A starch substrate solution (1 g/100 mL) was prepared using soluble starch (Merck KGaA, Darmstadt, Germany) in distilled water under gentle heating and continuous stirring until a clear, homogeneous solution was obtained. Lugol’s reagent was freshly prepared by dissolving 0.25 g of iodine (I_2_) in 10 mL of ethanol and combining this with asolution of 1 g of potassium iodide (KI) in 40 mL of distilled water. A stock of 2 M hydrochloric acid (HCl), prepared by appropriate dilution of 37% (*w/w*) HCl with deionized water, was used to quench the enzymatic reaction.

α-Amylase from *Aspergillus oryzae* (A8220; Sigma-Aldrich, St. Louis, MO, USA; ≥30 U/mg solid) was dissolved in the same phosphate buffer to yield a stock solution of 5 U/mL. In accordance with the manufacturer’s specification, one enzymatic unit was defined as the amount of enzyme that liberates 1.0 mg of maltose from starch in 3 min at pH 6.9 and 20 °C. Aliquots of 25 µL and 50 µL of this stock were used per reaction tube, providing approximately 0.14 and 0.28 U/mL during the enzymatic incubation (0.025 and 0.050 U/mL in the final 5.0 mL read-out volume), respectively.

Reaction mixtures with a final volume of 5.0 mL were assembled in glass tubes corresponding to the following conditions: a starch control, a reagent blank, two enzyme controls (25 and 50 µL of enzyme stock), and inhibitor blanks (IB) plus inhibitor tubes (IT) for each extract (E1–E3) and for the reference inhibitor, acarbose. Each tube received 600 µL of phosphate buffer; inhibitor-containing tubes were additionally supplemented with 100 µL of extract or acarbose working solution (2 mg/mL), corresponding to approximately 222–229 µg/mL during the enzymatic incubation (40 µg/mL in the final 5.0 mL read-out volume).

Enzyme (25 or 50 µL of stock, as designated) was then added and the mixtures were pre-incubated for 5 min at 37 °C using a temperature-controlled water bath (WTB, Memmert GmbH + Co. KG, Schwabach, Germany) to allow inhibitor–enzyme interaction prior to substrate addition. The enzymatic reaction was initiated by adding 150 µL of starch solution and allowed to proceed for 20 min at 37 °C. Hydrolysis was subsequently arrested by adding 2000 µL of 2 M HCl, followed by 50 µL of Lugol’s reagent for color development, and the final volume was adjusted to 5.0 mL with distilled water. Absorbance was recorded at 630 nm against the reagent blank using a UV–Vis spectrophotometer (UV-1900i, Shimadzu Corporation, Kyoto, Japan).

The percentage of α-amylase inhibition was calculated as:

(1) $$\%\;Inhibition=\frac{\left[\left(A_{IT}-A_{IB}\right)-A_{EC}\right]}{\left(A_{SC}-A_{EC}\right)}\times 100$$

where *A_SC_* is the absorbance of the substrate (starch) control incubated without enzyme (maximal residual starch staining), *A_EC_* is the absorbance of the uninhibited enzyme control at the same enzyme loading, *A_IT_* is the absorbance of the inhibitor-containing reaction tube, and *A_IB_* is the corresponding inhibitor blank (extract in buffer, without substrate or enzyme) used to correct for the intrinsic absorbance of the extracts. Under this formulation, 0% corresponds to full enzyme activity and 100% to complete inhibition.

### 4.7. Molecular Docking of the Notable Phytochemicals

In this study, molecular docking [32,33] was used to reveal possible binding modes and common interaction patterns of the selected plant phytochemicals against fungal and human α-amylase structures retrieved from the RCSB Protein Data Bank [34] (fungal *Aspergillus oryzae*: PDB IDs 3VX0 [35] and 7TAA [36,37]; human pancreatic α-amylase: PDB IDs 2QV4 [38] and 4GQR [39,40]). Ligands were retrieved as SMILES from PubChem [41]. Receptor protonation states were assigned using PDB2PQR [42] and PROPKA3 [43]. Ligand protonation and ionization states at pH 7.0 were assigned using Dimorphite-DL [44], and three-dimensional structures were generated and energy-minimized using Open Babel [45]. Rigid docking was performed with AutoDock Vina 1.2.7 [46,47] using a 22.5 × 22.5 × 22.5 Å grid centered on the catalytic pocket (3VX0: 12.266, 25.604, 29.919; 7TAA: 38.839, 39.756, 30.354; 2QV4: 12.385, 48.136, 26.209; 4GQR: 13.443, 15.135, 39.436). For each ligand, the number of modes was set to 20 and exhaustiveness to 64 (Supplementary Materials, Table S2).

Vina poses were rescored with GNINA 1.0 (CNN score/CNN affinity) [48] and RF-Score-v3 [49] implemented via ODDT [50], in order to reduce dependency on a single scoring function. Pose validity was assessed using PoseBusters [51] and RDKit [52] tests, and protein–ligand interaction profiles were annotated using PLIP [53]. Docking protocol performance was checked by redocking co-crystal ligands and evaluating symmetry-corrected RMSD using spyrmsd [54], as well as SuCOS scores, which provide a shape- and feature-based similarity comparison between two poses [55]. Complexes were visualized in UCSF ChimeraX [56], and 2D interaction diagrams were generated using the RDKit open-source cheminformatics software, version 2023.09.1 [52].

The four scoring metrics were interpreted as follows: (i) Vina score (kcal/mol): predicted binding free energy, with more negative values indicating stronger predicted binding; absolute values are not quantitatively comparable with experimental affinities. (ii) GNINA CNN score (range 0–1): the convolutional neural network’s classification of pose validity, where higher values indicate higher confidence. (iii) GNINA CNN affinity: a deep-learning-derived predicted binding affinity expressed as −log(Kd) in approximate pK units. (iv) RF-Score-v3: a random-forest-based predicted binding affinity, also in approximate pK units. Higher values for CNN affinity and RF-Score-v3 indicate stronger predicted binding. The use of multiple orthogonal scoring functions is intended to mitigate over-reliance on any single function.

Although the inhibition assay used in the present work was based exclusively on the fungal α-amylase from *A. oryzae*, docking was deliberately extended to two human pancreatic α-amylase structures (PDB IDs 2QV4 and 4GQR) in addition to the fungal targets (3VX0 and 7TAA). This cross-species design was motivated by the strong sequence and active-site conservation at the catalytic triad (Asp206/Asp297/Glu230 in 7TAA correspond to Asp197, Asp300, Glu233 in human enzymes [57,58]) and was intended to evaluate whether the binding-site recognition observed for the quantified phytochemicals would generalize across the two enzyme classes. The human α-amylase docking results should therefore be interpreted strictly as a hypothesis-generating in silico extrapolation rather than as direct evidence of activity on the mammalian enzyme; experimental confirmation using porcine pancreatic or recombinant human α-amylase is necessary for follow-up studies.

The 22.5 Å grid edge length was empirically validated to accommodate all five ligands in their extended conformations, with the largest ligand (apigenin-7-*O*-glucoside) showing no poses with atoms within 2 Å of the grid boundary across the four receptors tested.

PoseBusters was applied with default chemical and physical validity checks (bond lengths and angles, planarity of aromatic rings, chirality preservation, internal energy, and intramolecular clashes). Poses that achieved higher scores or a larger number of interactions but failed these validity checks, or displayed severe steric clashes, were discarded during visual inspection and were not considered for final selection. Additional pose-selection metrics for the selected top poses are summarized in Supplementary Materials (Table S4).

Prior to phytochemical docking, crystallographic water molecules, co-crystallized ligands, and non-structural ions/molecules were removed, while structural Ca^2+^ was retained. Polar hydrogens were added and receptor protonation states were assigned at pH 7.0 using PDB2PQR [42] and PROPKA3 [43]. A separate receptor-preparation branch retaining catalytically relevant waters was used only for co-crystal redocking validation, as described in the Supplementary Materials.

Physicochemical, pharmacokinetic (ADME) and drug-likeness descriptors of the five quantified markers were predicted using SwissADME [59] (http://www.swissadme.ch/; accessed on 20 September 2026) (Figure S5); toxicity endpoints were not predicted.

### 4.8. Statistical Analysis and Computational Processing

Each preparation (E1–E3) was extracted once, and reported values are presented as mean ± SD of three analytical replicates (*n* = 3) obtained from the corresponding extract. Accordingly, these replicates were used to characterize analytical precision and were not treated as independent extraction or biological replicates for inferential between-preparation testing. Between-preparation differences are therefore presented descriptively. Relationships between marker contents and apparent α-amylase inhibition were only visualized as scatter plots (Figure 1); because only three preparations were available (df = 1 for a linear fit), no regression models, correlation coefficients or *p*-values were computed or interpreted. In silico computations were performed on a Linux system within a Python 3.11 conda environment; docking and related post-processing workflows are described in Section 4.7.

## 5. Conclusions

This exploratory study compared three preparations of *Arum maculatum* designed to mirror the dried, fresh and boiled states in which the plant is handled. All three extracts reduced the end-point starch–iodine signal of *Aspergillus oryzae* α-amylase more strongly than the assay-internal acarbose control at the same nominal mass concentration, and the decoction (E3) gave the highest apparent inhibition at the higher enzyme loading. The five phenolic markers previously quantified in these extracts did not follow this pattern and, at no more than about 2 µM during the incubation, are unlikely to account for it; docking placed them in the catalytic cleft but provides structural hypotheses only. The main conclusion supported by the data is therefore that the way *A. maculatum* is processed and extracted is associated with different apparent α-amylase inhibition, whereas the constituents responsible remain unidentified. Because each preparation was represented by a single extract, inhibition was measured at one concentration with one end-point method, and no safety-related endpoint was assessed, these findings are descriptive and do not support any functional-food or dietary use. Independent replicate preparations, dose–response and orthogonal assays with mammalian enzymes, untargeted LC–MS/MS with bioassay-guided fractionation, and a quantitative oxalate, raphide and toxicity assessment of the processed material are the necessary next steps.

## Figures and Tables

**Table 1:** Phenolic marker concentrations (mg/g extract, mean ± standard deviation (SD) of *n* = 3 analytical replicates of a single extract per preparation). The mean values were previously reported in [12]; the SDs of the analytical replicates are reported here for the first time.

Marker (mg/g Extract)	E1 (Dried)	E2 (Fresh)	E3 (Decoction)
Gallic acid	1.459 ± 0.038	0.563 ± 0.016	1.092 ± 0.029
Chlorogenic acid	0.977 ± 0.026	0.880 ± 0.024	0.790 ± 0.022
Ferulic acid	n.d. *	0.653 ± 0.018	0.186 ± 0.007
Apigenin-7-*O*-glucoside	0.052 ± 0.003	n.d. *	n.d. *
Quercetin	0.541 ± 0.016	0.622 ± 0.018	0.128 ± 0.005

* n.d.: not detected.

**Table 2:** Apparent α-amylase inhibition (%, mean ± SD of three analytical replicates, *n* = 3) at the same nominal concentration for all samples (E1–E3 and acarbose; 40 µg/mL in the 5.0 mL read-out volume, ≈222–229 µg/mL during incubation).

Sample	Enzyme 25 µL	Enzyme 50 µL
E1	81.09 ± 1.87	75.13 ± 2.14
E2	77.50 ± 2.03	79.94 ± 1.76
E3	79.94 ± 1.92	90.80 ± 1.58
Acarbose	67.28 ± 1.65	68.15 ± 1.72

**Table 3:** Selected top pose of each phytochemical for each α-amylase receptor, with co-crystal redocking scores as reference benchmarks. “Catalytic interactions” lists catalytic residues in polar contact with the ligand, including borderline contacts (donor–acceptor distance > 3.5 Å or donor–H–acceptor angle < 110°; see Section 2.4 and Supplementary Materials). ABC, QV4 and MYC are the co-crystallized ligands of 7TAA, 2QV4 and 4GQR, respectively. The upper section shows redock validation scores for these ligands; the lower section shows docking results for the five phenolic phytochemicals. Pose-selection criteria are described in Section 4.7. The reference scores are an approximate benchmark only: the redocked poses of the co-crystal ligands, and hence their scores, depend on whether crystallographic waters are retained (Supplementary Materials, Table S3), whereas all phytochemicals were docked into desolvated receptors.

	Receptor	Chemical	Vina Score (kcal/mol)	CNN Score	CNN Affinity	RF-Score-v3	Catalytic Interactions
Co-crystal (redock) reference scores	7TAA	ABC	−13.77	0.80	7.90	5.95	Asp206, Asp297, Glu230
	2QV4	QV4	−12.68	0.50	7.80	6.18	Asp197, Asp300, Glu233
	4GQR	MYC	−9.45	0.42	4.34	5.57	Asp197, Asp300, Glu233
Phytochemical docking scores	3VX0	Apigenin-7-*O*-glucoside	−8.76	0.05	4.60	5.77	Asp297
	3VX0	Chlorogenic acid	−8.18	0.61	5.19	5.81	Asp206, Asp297
	3VX0	Ferulic acid	−5.66	0.48	4.19	5.08	Asp206, Glu230
	3VX0	Gallic acid	−6.08	0.74	4.54	4.12	Asp206, Asp297
	3VX0	Quercetin	−9.27	0.36	4.53	5.66	Asp206, Asp297, Glu230
	7TAA	Apigenin-7-*O*-glucoside	−10.14	0.14	5.50	6.16	Asp206, Asp297, Glu230
	7TAA	Chlorogenic acid	−9.17	0.42	4.59	5.72	Asp206, Glu230
	7TAA	Ferulic acid	−5.64	0.62	4.24	5.09	Asp297, Glu230
	7TAA	Gallic acid	−6.16	0.82	4.08	4.16	Asp206, Asp297
	7TAA	Quercetin	−9.36	0.26	4.25	5.64	Asp206, Asp297
	2QV4	Apigenin-7-*O*-glucoside	−10.66	0.49	5.96	6.14	Asp197, Asp300, Glu233
	2QV4	Chlorogenic acid	−8.31	0.20	4.74	5.96	Asp300, Glu233
	2QV4	Ferulic acid	−6.19	0.53	4.32	4.98	Asp197, Asp300
	2QV4	Gallic acid	−6.74	0.59	3.85	4.07	Asp197, Asp300, Glu233
	2QV4	Quercetin	−10.36	0.71	5.22	5.70	Asp197, Glu233
	4GQR	Apigenin-7-*O*-glucoside	−9.04	0.15	4.92	6.19	Asp197, Asp300
	4GQR	Chlorogenic acid	−7.89	0.47	4.64	5.71	Asp197, Glu233
	4GQR	Ferulic acid	−6.09	0.44	4.48	5.16	Asp197, Glu233
	4GQR	Gallic acid	−5.80	0.69	4.10	4.18	Asp197, Asp300
	4GQR	Quercetin	−9.53	0.58	4.80	5.74	Asp197, Glu233

## Data Availability

The phenolic marker data (Table 1, Table S1) were previously published in [12]; the chromatograms in Figure S1 come from the same analyses. The raw data supporting the conclusions of this article will be made available by the authors on request.

## Supplementary Materials

The following supporting information can be downloaded at: https://www.mdpi.com/article/10.3390/molecules31213994/s1, Figure S1: HPLC–PDA chromatograms of E1–E3 (from the analyses reported in [12]); Table S1: HPLC–PDA calibration parameters (previously reported in [12]); Table S2: AutoDock Vina docking parameters and grid definitions; Table S3: Redocking RMSD and SuCOS comparisons; Table S4: Selected docking poses and additional metrics; Table S5: Phenolic marker contents of E2 and E3 per gram of fresh material; Figures S2–S4: 3D and 2D docking visualizations for 3VX0, 2QV4, and 4GQR; Figure S5: physicochemical, ADME and drug-likeness properties; Figure S6: docking-affinity heat map; Figure S7: comparative radar plot. References [12,34,35,36,37,38,39,40,41,42,43,44,45,46,47,48,49,50,51,52,53,54,55,56,58] are cited in the Supplementary Materials.

## Abbreviations

The following abbreviations are used in this manuscript:

ADME	Absorption, distribution, metabolism and excretion
CNN	Convolutional neural network
CUPRAC	Cupric ion reducing antioxidant capacity
DNS	3,5-Dinitrosalicylic acid
DPPH	2,2-Diphenyl-1-picrylhydrazyl
E1	Dried plant–ethanol:water extract
E2	Fresh plant–ethanol:water extract
E3	Decoction extract
HPLC–PDA	High-performance liquid chromatography with photodiode array detection
IB	Inhibitor blank
IC_50_	Half-maximal inhibitory concentration
IT	Inhibitor tube
*K_i_*	Inhibition constant
*K_m_*	Michaelis constant
LC–MS	Liquid chromatography–mass spectrometry
LOD	Limit of detection
LOQ	Limit of quantification
PDA	Photodiode array
PDB	Protein Data Bank
PLIP	Protein–Ligand Interaction Profiler
RCSB	Research Collaboratory for Structural Bioinformatics
RMSD	Root-mean-square deviation
SD	Standard deviation
SMILES	Simplified Molecular Input Line Entry System
SuCOS	Shape- and feature-based overlap score
