# Supplementary material for: Traditional Processing of Arum maculatum L. and Apparent α-Amylase Inhibition: An Exploratory In Vitro and In Silico Study
Source: Molecules. 2026 Nov 5;31(21):3994. doi: 10.3390/molecules31213994 (PMC13764867; doi:10.3390/molecules31213994)
Supplement: Supplementary file 1 [file molecules-31-03994-s001.zip › molecules-4624500-supplementary.pdf]

Supplementary Materials

Traditional Processing of *Arum maculatum* L. and Apparent α-Amylase Inhibition: An Exploratory In Vitro and In Silico Study

Zafer Ömer Özdemir, Ezgi Keskin, Mustafa Niğde, Duygu Mısırlı, Ebru Özdemir Nath and Mahfuz Elmastaş

a

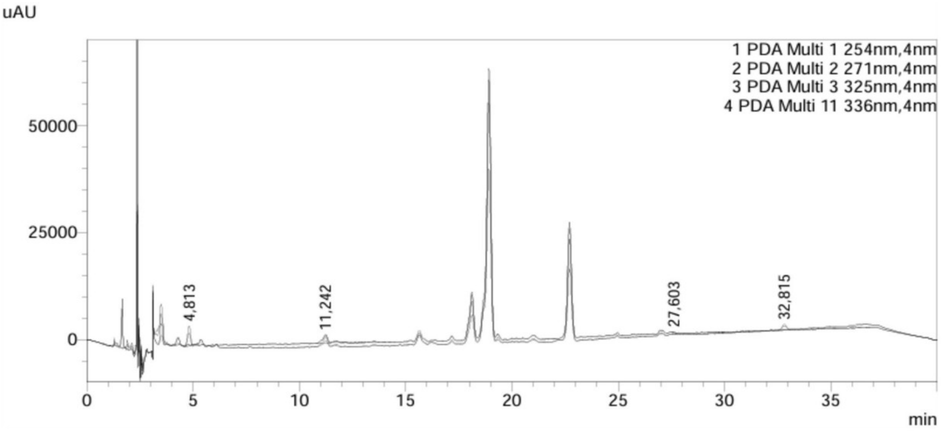

b

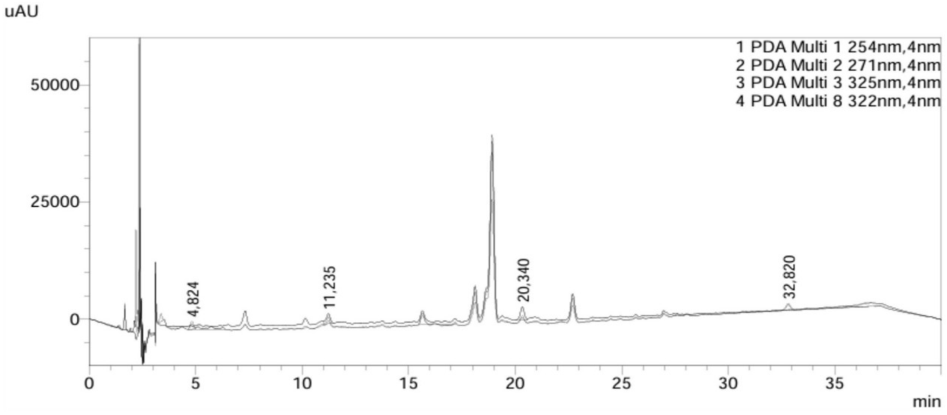

c

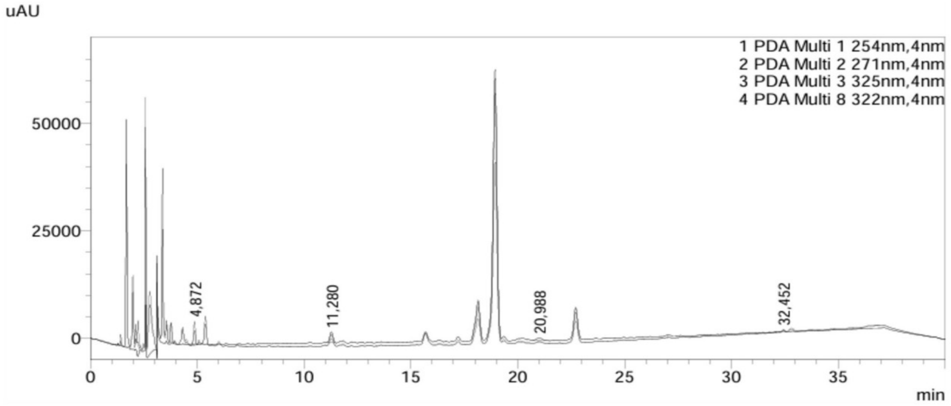

Figure S1. HPLC–PDA chromatograms of extracts: (a) E1, ethanol:water extract prepared from dried *A. maculatum* plant material; (b) E2, ethanol:water extract prepared from fresh *A. maculatum* plant material; (c) E3, decoction (aqueous) extract prepared from fresh *A. maculatum* plant material. The chromatograms were recorded in the same HPLC–PDA analyses whose quantitative results (Table 1 of the main text; Table S1) were previously reported in Keskin et al. [S25]. They are shown as exported from the instrument software at the quantification wavelengths listed in Table S1 (254, 271 and 325 nm, plus 336 nm for E1 and 322 nm for E2 and E3)

and therefore differ in detection wavelengths and layout from Figure 1 of [S25]. Numbers above the peaks are retention times in minutes (decimal comma as exported by the software).

Table S1. HPLC–PDA calibration parameters (previously reported in Keskin et al. [S25] and reproduced here for completeness).

Compound	RT* (min)	λmax* (nm)	Calibration equation	R^2	LOD* (mg/L)	LOQ* (mg/L)
Gallic acid	4.352	271	y = 29799.9x + 6494.60	0.9995	0.744	2.255
Chlorogenic acid	12.073	325	y = 28066.0x + 25870.2	0.9990	1.369	4.148
Ferulic acid	20.971	322	y = 46665.0x − 14606.2	0.9928	1.387	4.202
Apigenin-7-*O*-glucoside	27.574	336	y = 39321.9x + 1685.18	0.9993	0.730	2.211
Quercetin	32.008	254	y = 26403.4x + 1558.71	0.9997	1.272	3.856

* RT: Retention time; λmax: Maximum absorbance wavelength; LOD: Limit of detection; LOQ: Limit of quantification. Calibration was performed using external standards at multiple concentrations. Column: InertSustain Phenylhexyl (150 × 4.6 mm, 3 μm; GL Sciences Inc., Tokyo, Japan); Column temperature: 30 °C; Flow rate: 1 mL/min. The complete gradient program, injection volume and quantification wavelengths are given in Section 4.5 of the main text.

1. Protein Structure Selection and Preparation

Four α-amylase crystal structures were retrieved from the RCSB Protein Data Bank [S1]:

- 3VX0: *Aspergillus oryzae* α-amylase (fungal, apo form) [S2].
- 7TAA: *Aspergillus oryzae* α-amylase (fungal, ligand-bound; co-crystal: acarbose derivative) [S3, S4].
- 2QV4: Human pancreatic α-amylase (ligand-bound; co-crystal: acarbose-derived inhibitor) [S5, S6].
- 4GQR: Human pancreatic α-amylase (ligand-bound; co-crystal: myricetin) [S7, S8].

Receptor preparation (water removal, retention of structurally relevant ions, hydrogen assignment, and protonation at pH 7.0 with PDB2PQR [S9] / PROPKA3 [S10]) is described in the main manuscript (Section 4.7).

2. Ligand Preparation

Ligand structures (gallic acid, chlorogenic acid, ferulic acid, apigenin-7-*O*-glucoside, and quercetin) were retrieved as SMILES from PubChem [S11], protonation and ionization at pH 7.0 were assigned with Dimorphite-DL [S12], and 3D coordinates were generated and energy-minimized in Open Babel [S13] before conversion to PDBQT format. Full method details and reference list are given in the main manuscript (Section 4.7).

3. Docking Parameters

Docking was performed with AutoDock Vina 1.2.7 [S14, S15] using the parameters summarized in Table S2 below; the catalytic-pocket grid coordinates are also reported in the main manuscript (Section 4.7).

Table S2. AutoDock Vina docking parameters and grid definitions.

Parameter	3VX0	7TAA	2QV4	4GQR
Grid center (x, y, z)	12.266, 25.604, 29.919	38.839, 39.756, 30.354	12.385, 48.136, 26.209	13.443, 15.135, 39.436
Grid size (Å)	22.5 × 22.5 × 22.5	22.5 × 22.5 × 22.5	22.5 × 22.5 × 22.5	22.5 × 22.5 × 22.5
Exhaustiveness	64	64	64	64
Number of modes	20	20	20	20

4. Pose Rescoring and Validation

Vina poses were rescored using multiple scoring functions to reduce single-method dependency:

- GNINA 1.0 [S16]: CNNscore and CNNaffinity metrics.
- RF-Score-v3: Via ODDT (Open Drug Discovery Toolkit) [S17, S18].

Pose quality was assessed using:

- PoseBusters [S19]: Chemical validity checks.
- RDKit [S20]: Geometric validation.
- PLIP (Protein-Ligand Interaction Profiler) [S21]: Interaction annotation.

Docking protocol performance was validated by redocking co-crystallized ligands. Symmetry-corrected RMSD values were calculated using spyrmsd [S22], and SuCOS [S23] was used as a complementary shape and feature similarity measure. For each receptor, two receptor-preparation branches were generated: in the first one all crystallographic waters were removed along with every ion and molecule except structural calcium. These were used for the docking study of phytochemicals. The second set was only used for redocking crystallographic ligands to the original structures to evaluate docking method accuracy. This set was the same as the first one except water molecules were retained within 6 Å of the catalytic center and within 3.5 Å of the protein. Therefore, the relevant portions of the original water network were maintained to better bind the original ligands ABC, QV4 and MYC to their receptors. Table S3 shows the results.

Table S3. Redocking RMSD and SuCOS comparisons.

Ligand	Receptor	sc-RMSD* with desolvated receptor	sc-RMSD* with retained water	SuCOS* with desolvated receptor	SuCOS* with retained water
ABC	7TAA	6.78 Å	0.69 Å	0.70	0.88
QV4	2QV4	7.75 Å	1.16 Å	0.67	0.88

Ligand	Receptor	sc-RMSD* with desolvated receptor	sc-RMSD* with retained water	SuCOS* with desolvated receptor	SuCOS* with retained water
MYC	4GQR	2.34 Å	0.90 Å	0.90	0.98

* RMSD: Root-mean-square deviation (symmetry-corrected); SuCOS: Shape and feature similarity score. RMSD values < 2.0 Å indicate acceptable pose reproduction in most cases, while higher SuCOS generally indicates higher shape and pharmacophoric feature similarity. As expected, retained water molecules helped the ligands bind similarly to the original poses.

5. Tiered Pose Selection Methodology

The approach to determining the top poses was the application of a tiered, multi-parameter evaluation method. Each tier was intended as a further refinement of the previous one. First, the poses that did not interact with any of the catalytic residues or the ones that did not pass pose quality checks or have severe clashes were eliminated, however good scores they might present. Second, the compounds were ranked according to the primary metrics, namely their Vina score, CNN-score, CNN-affinity, and RF-score-v3 to see if there is a consensus rank for any. Third, the interaction data, namely key catalytic interactions and interaction properties such as the number and diversity of interactions and presence of unsatisfied acceptors/donors were utilized for refining the ranking. Then, if the ranks were still very close, the poses were also ranked according to the remaining metrics—buriedness percentage and ligand efficiency (LE)—for deciding between very closely ranked poses. However, this step was rarely needed. Buriedness percentages were not used as a filter; rather, they were used as comparative metrics to assess whether ligand exposure differed substantially from that of the original redocked ligands. Buriedness percentages of these ligands, namely ABC, MYC and QV4, were between 71–78%, not extremely different than the study ligands. Lastly, a visual check was performed to confirm the findings.

6. Cross-Receptor Pose Similarity and Extended Analysis

SuCOS [S23], along with interaction fingerprint analysis, was also applied to determine cross-receptor similarity of phytochemicals used in this study for both inter- and intra-organism level. The main benefit of this approach was the evaluation of binding mode differences across ligands and receptors without just relying on RMSD data. SuCOS and Jaccard similarity analysis could provide valuable insight in these cases. To achieve this, catalytic pockets were aligned between receptors, and the relevant residues were overlapped as a first step. In the second step, to enable cross-receptor comparison, residue identifiers were projected into a common reference residue space via a carefully prepared pocket residue mapping. Then, feature and geometry similarities between poses were calculated as described in the literature. Interaction fingerprint similarity between two poses was computed with the Jaccard index, $J(A,B) = |A \cap B| / |A \cup B|$. To obtain cross-receptor scores per pose, the best matches combining SuCOS and interaction similarity were utilized via:

$$Score_{combined} = ½\, S_{SuCOS} + ½\, J(A,B) \qquad (1)$$

This score prioritizes matches that are simultaneously similar in 3D pose geometry and interaction pattern, rather than selecting the maximum data of either alone. Additionally, ligand efficiency (LE = |Vina score| / heavy atom count), interaction diversity, total interaction count, and buriedness percentage metrics were obtained to interpret the scores of different poses.

7. Cross-Receptor Binding Mode Comparison

Across the four receptors, same-ligand comparisons of SuCOS and Jaccard values are informative for distinguishing intra- from inter-organism consistency; the dataset-wide best matches of the selected top poses are listed separately in Table S4. Within the human enzymes (2QV4 and 4GQR), SuCOS values are moderate to high (~0.49–0.84) while Jaccard values remain low to moderate (~0.12–0.48), indicating that geometrically similar ligand placement can be retained even when the residue-level interaction network is less strongly conserved (combined scores ~0.38–0.63).

Within the fungal enzymes (3VX0 and 7TAA), conservation appears stronger overall: SuCOS values span ~0.48–0.95 and Jaccard values span ~0.36–1.00, with several ligands (quercetin, gallic acid, chlorogenic acid, and ferulic acid) approaching near-complete interaction-fingerprint overlap and combined scores up to ~0.98. Apigenin-7-*O*-glucoside is a partial exception, with a distinctly asymmetric 7TAA–3VX0 comparison, indicating that fungal conservation is ligand-dependent rather than absolute.

Cross-organism comparisons remain weaker than intra-organism ones, as expected. SuCOS values span ~0.25–0.71 and Jaccard values 0.07–0.67, with same-ligand combined scores (~0.17–0.57) generally lower than the intra-organism values. In the dataset-wide search (Table S4), only the gallic acid poses from the human structures found their best match in the fungal enzyme 3VX0 (0.71 and 0.59). These results are most consistent with partial mode conservation accompanied by receptor-specific remodeling of the polar and ionic interaction network, rather than strictly conserved binding modes across fungal and human enzymes.

The pattern also suggests that geometric resemblance and interaction-pattern conservation are not interchangeable: some intra-human comparisons show favorable SuCOS despite modest Jaccard overlap, while several fungal comparisons show high Jaccard with only moderate SuCOS. Combined-score interpretation is therefore preferable to either metric alone. Overall, high cross-receptor similarity should be read as limited evidence of partially conserved binding within the sampled pose space, rather than proof of a single conserved pose. Definitive conclusions on cross-organism binding-mode conservation would require molecular dynamics simulations, explicit water treatment, broader ensemble docking, and experimental confirmation.

Table S4. Selected docking poses and additional metrics

Receptor	Phytochemical	Top pose interaction diversity / Total interactions	Top pose total minor clashes	Top pose LE (vina)	Top pose Buriedness %	Best cross-receptor combined score in dataset	Best match for cross-receptor analysis
3VX0	Quercetin	3 / 11	1	0.42	79.9	0.65	7TAA
3VX0	Chlorogenic acid	4 / 8	2	0.33	80.3	0.67	7TAA
3VX0	Gallic acid	2 / 5	0	0.51	83.2	0.63	7TAA
3VX0	Ferulic acid	2 / 4	0	0.40	79.0	0.78	7TAA
3VX0	Apigenin-7-*O*-glucoside	3 / 5	0	0.28	65.5	0.50	7TAA
7TAA	Chlorogenic acid	3 / 6	0	0.37	81.6	0.80	3VX0
7TAA	Quercetin	2 / 5	2	0.43	83.7	0.90	3VX0
7TAA	Gallic acid	3 / 7	0	0.51	84.0	0.74	3VX0
7TAA	Ferulic acid	2 / 4	0	0.40	81.6	0.78	3VX0
7TAA	Apigenin-7-*O*-glucoside	3 / 10	0	0.33	71.3	0.48	3VX0

Receptor	Phytochemical	Top pose interaction diversity / Total interactions	Top pose total minor clashes	Top pose LE (vina)	Top pose Buriedness %	Best cross-receptor combined score in dataset	Best match for cross-receptor analysis
2QV4	Quercetin	3 / 10	1	0.47	77.2	0.82	4GQR
2QV4	Chlorogenic acid	3 / 6	1	0.33	78.6	0.49	4GQR
2QV4	Ferulic acid	2 / 4	0	0.44	84.4	0.54	4GQR
2QV4	Gallic acid	2 / 7	0	0.56	84.5	0.71	3VX0
2QV4	Apigenin-7-*O*-glucoside	3 / 9	1	0.34	71.0	0.66	4GQR
4GQR	Quercetin	3 / 11	1	0.43	77.5	0.82	2QV4
4GQR	Chlorogenic acid	3 / 7	1	0.32	79.7	0.68	2QV4
4GQR	Ferulic acid	3 / 5	0	0.43	84.4	0.84	2QV4
4GQR	Gallic acid	2 / 3	0	0.48	83.0	0.59	3VX0
4GQR	Apigenin-7-*O*-glucoside	2 / 6	0	0.29	70.4	0.52	2QV4

Note: Best cross-receptor combined score in dataset: highest combined score (½ SuCOS + ½ Jaccard) obtained when the selected top pose was compared with all docked poses of all ligands in the other receptors; the receptor containing the best-matching pose is given in the last column. Because this search is not restricted to the same ligand or to the selected top poses, these values can differ from the same-ligand ranges reported in Section 7.

Table S5. Phenolic marker contents of E2 and E3 per gram of fresh material

Marker	E2 (μg/g fresh material)	E3 (μg/g fresh material)	E3/E2 ratio
Gallic acid	5.83	42.01	7.2
Chlorogenic acid	9.11	30.39	3.3
Ferulic acid	6.76	7.16	1.1
Apigenin-7-O-glucoside	n.d.	n.d.	—
Quercetin	6.44	4.92	0.76

Values were calculated as mg/g extract (Table 1) × lyophilized extract yield (1.52 g for E2; 5.65 g for E3) / 146.85 g fresh material. n.d.: not detected. Because E2 and E3 were prepared from identical amounts of the same fresh material, these values place the two preparations on a common fresh-weight basis; however, the preparations also differed in solvent composition and volume (300 mL ethanol:water 80:20 vs. 500 mL water), so the E3/E2 ratios reflect the combined preparation conditions and not the effect of heating alone. The mg/g extract values used in this calculation were previously reported in [S25]; E1 (prepared from dried material of undetermined initial moisture content) cannot be included on this basis.

8. Extended Docking Visualizations

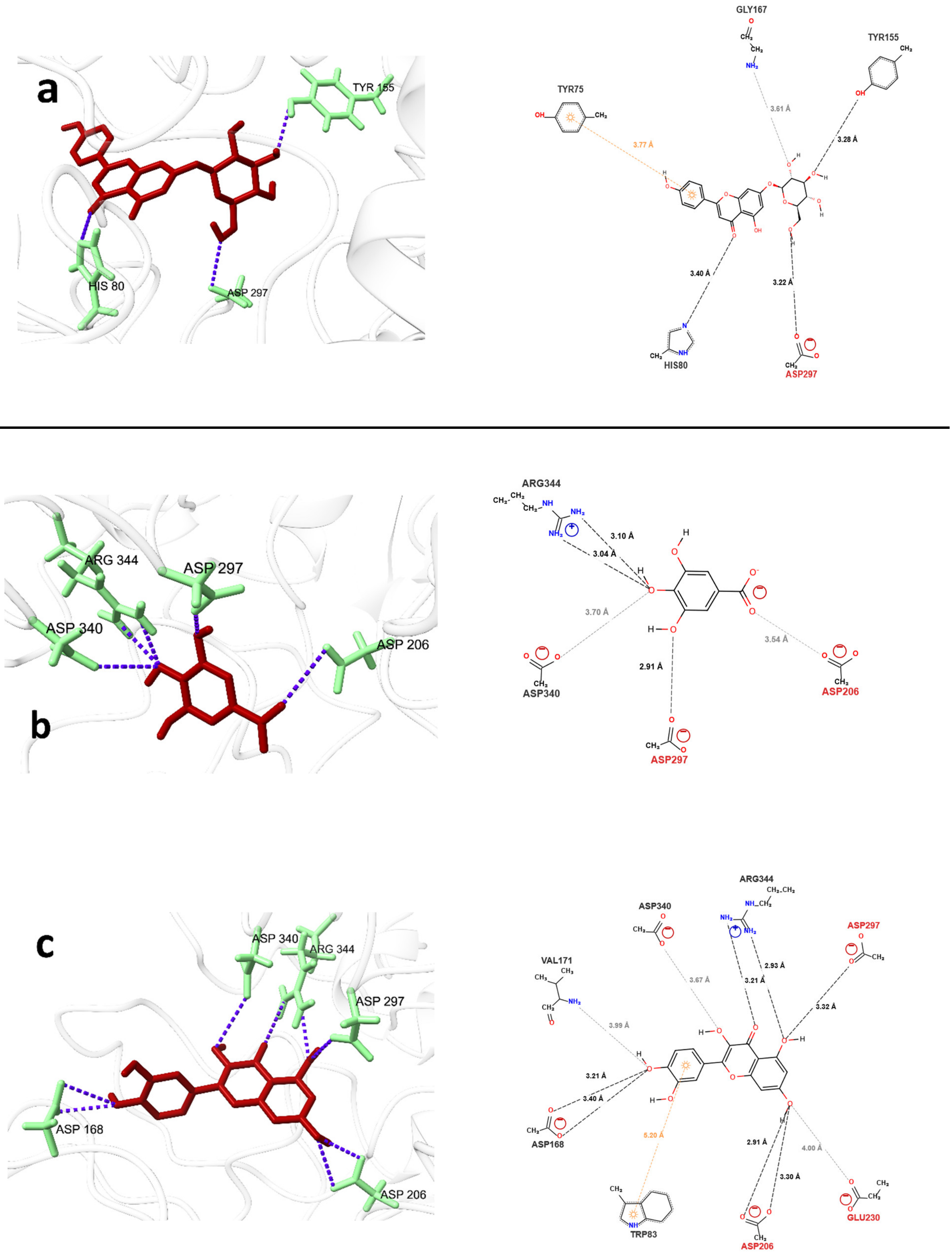

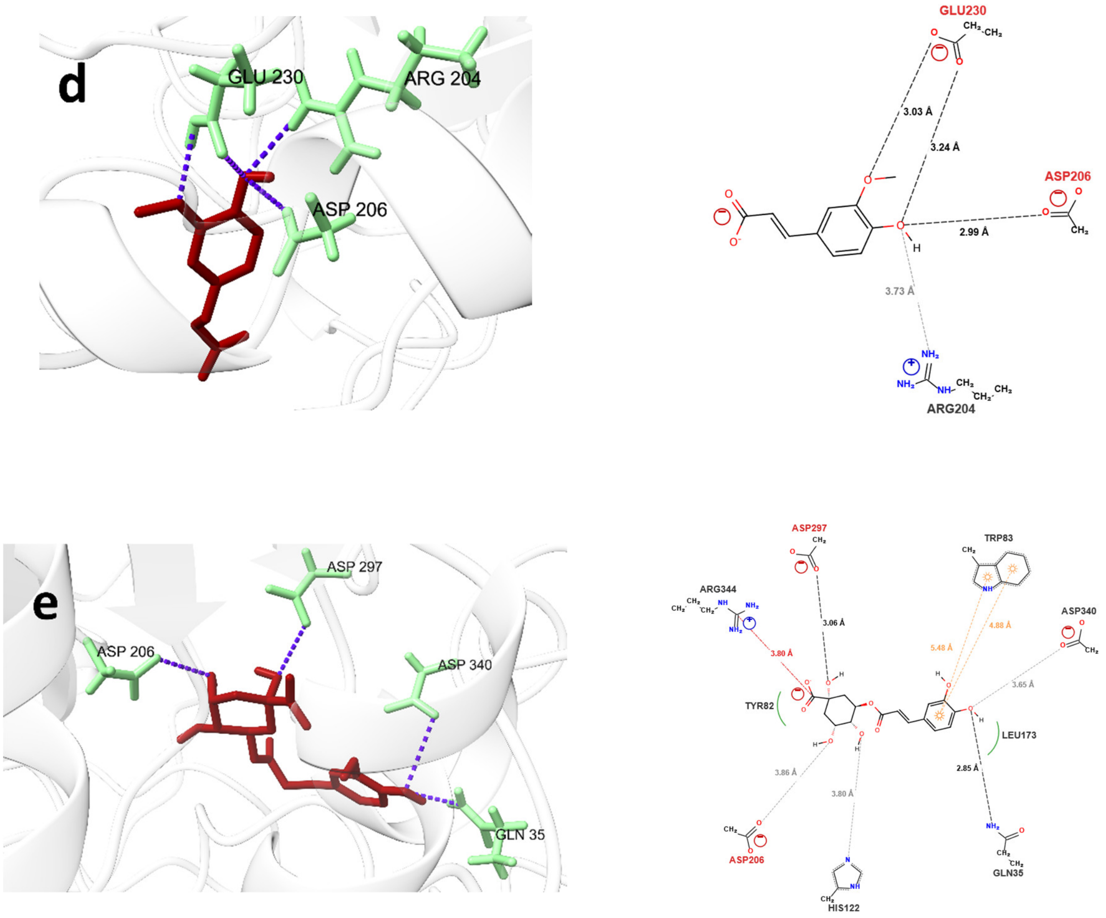

Figure S2. 3D and 2D visualizations of the top complexes for 3VX0. From top to bottom: (a) apigenin-7-*O*-glucoside, (b) gallic acid, (c) quercetin, (d) ferulic acid, and (e) chlorogenic acid. The 3D depiction is shown only with the most important interacting residues for clarity.

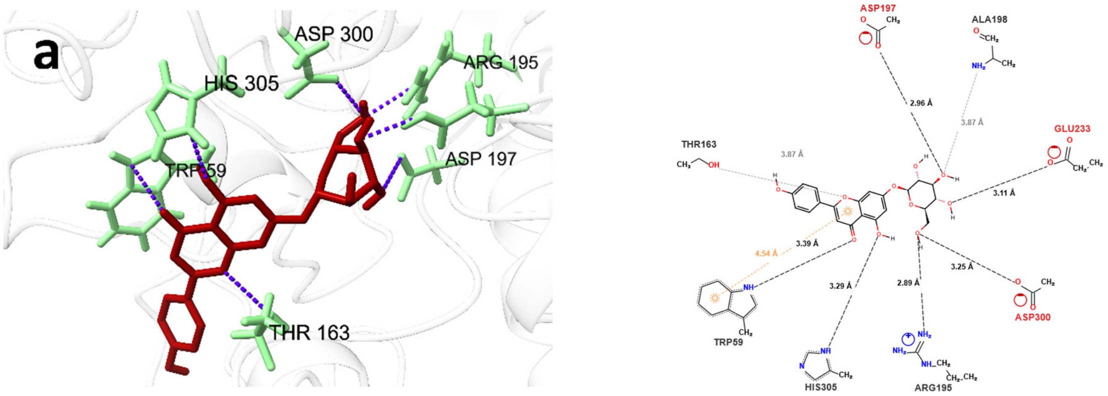

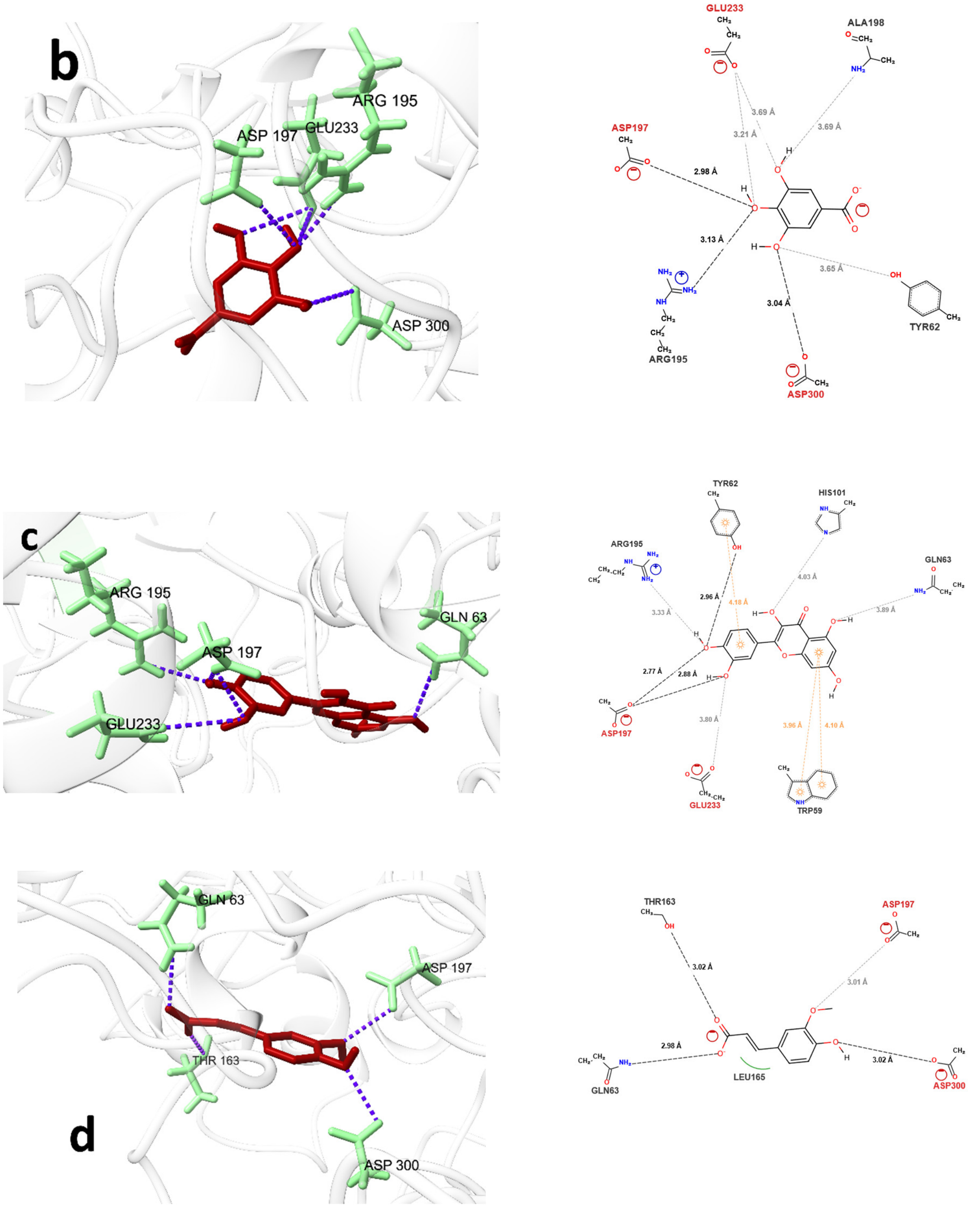
b
ARG 195
ASP 197
GLU233
ASP 300
GLU233
ALA198
ASP197
3.69 Å
3.21 Å
3.69 Å
2.98 Å
3.13 Å
3.65 Å
3.04 Å
ARG195
TYR62
ASP300
c
ARG 195
GLN 63
ASP 197
GLU233
TYR62
HIS101
ARG195
GLN63
2.96 Å
4.18 Å
4.03 Å
3.33 Å
3.89 Å
2.77 Å
2.88 Å
3.80 Å
3.96 Å
4.10 Å
ASP197
GLU233
TRP59
GLN 63
ASP 197
THR 163
ASP 300
d
THR163
ASP197
3.02 Å
3.01 Å
2.98 Å
3.02 Å
GLN63
LEU165
ASP300

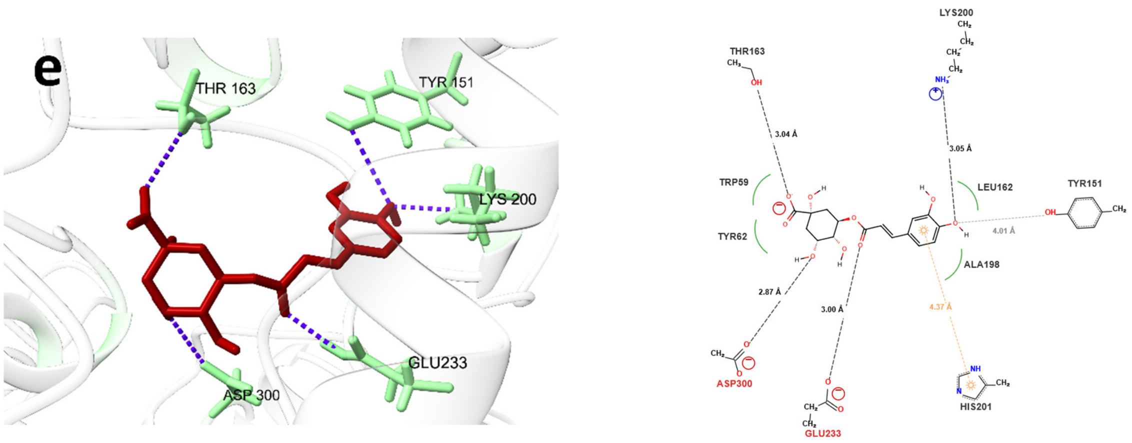

Figure S3. 3D and 2D visualizations of the top complexes for 2QV4. From top to bottom: (a) apigenin-7-*O*-glucoside, (b) gallic acid, (c) quercetin, (d) ferulic acid, and (e) chlorogenic acid. The 3D depiction is shown only with the most important interacting residues for clarity.

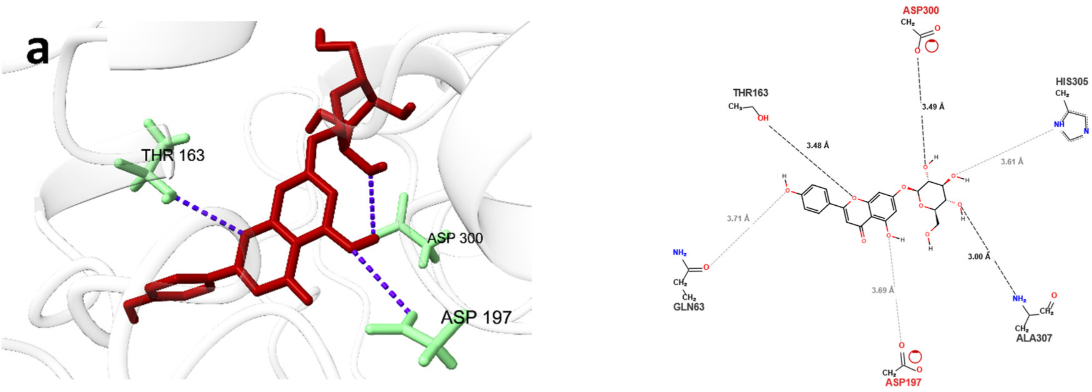

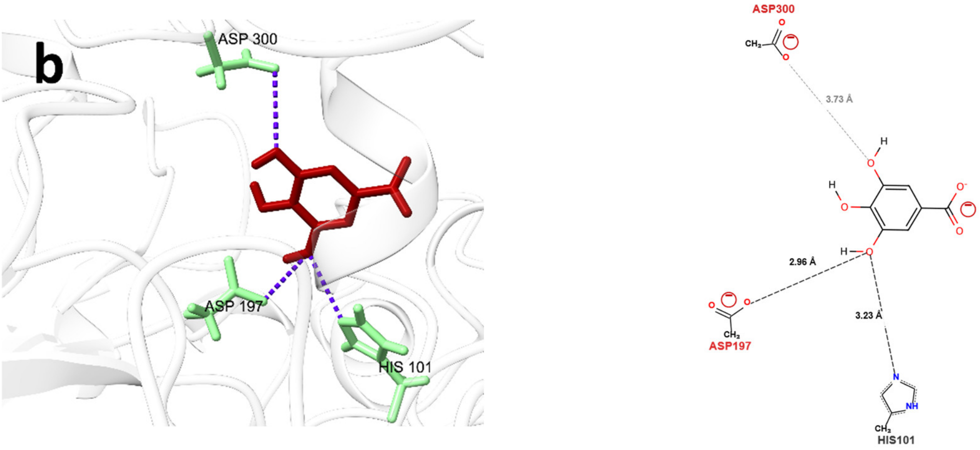

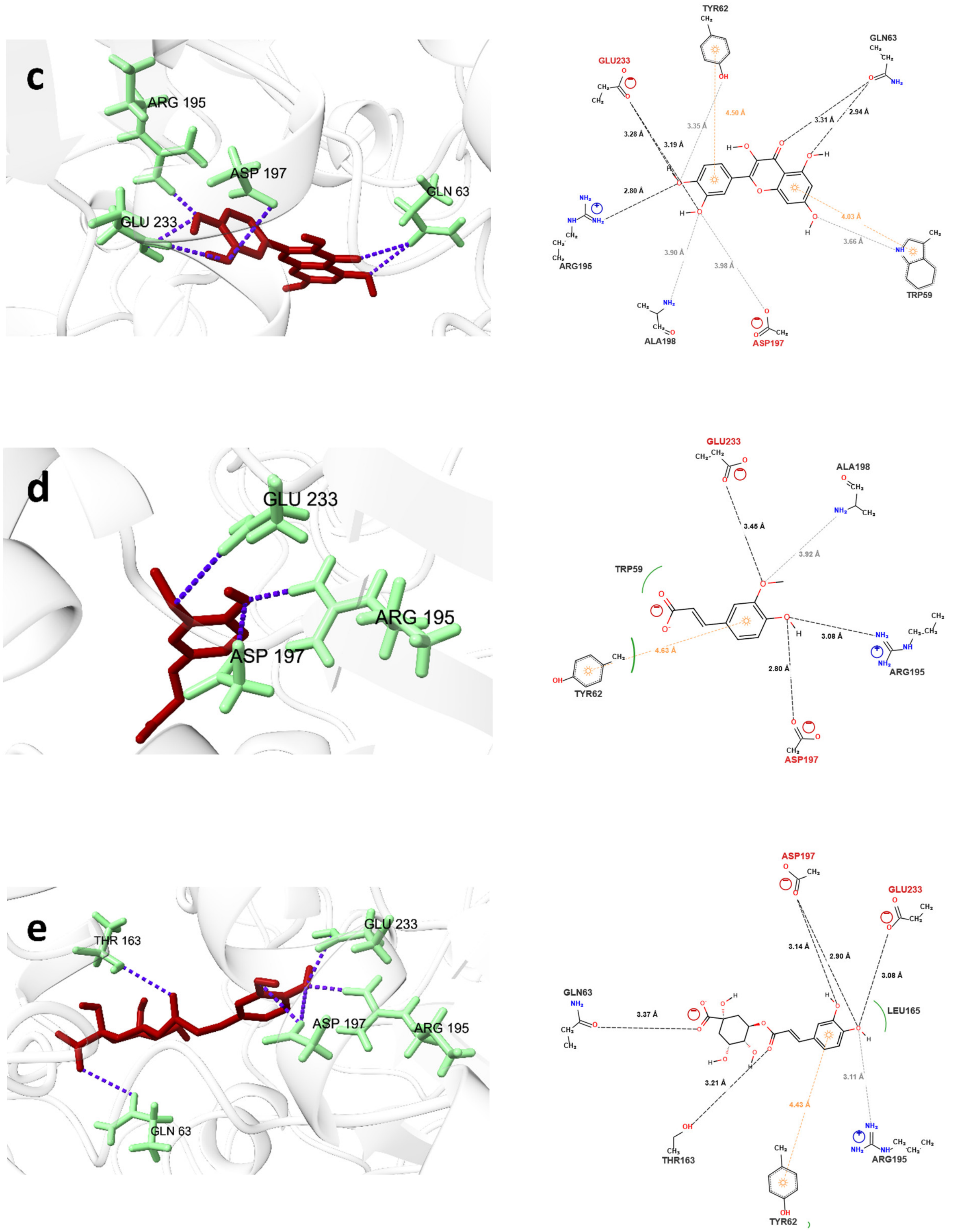

Figure S4. 3D and 2D visualizations of the top complexes for 4GQR. From top to bottom: (a) apigenin-7-*O*-glucoside, (b) gallic acid, (c) quercetin, (d) ferulic acid, and (e) chlorogenic acid. The 3D depiction is shown only with the most important interacting residues for clarity.

Three-dimensional complex visualizations were prepared using UCSF ChimeraX [S24], and two-dimensional interaction diagrams were generated using RDKit [S20].

For the 3VX0 poses, the interaction pattern is distributed across an acidic/polar pocket in which Glu230, Asp206, Asp297, and nearby residues contribute in a ligand-dependent manner. Ferulic acid is seen in the figure in the least complex arrangement, showing connections against Glu230/Arg204 and near Asp206; correspondingly, a few polar contacts to Glu230 at 3.03–3.24 Å with 106.9–140.8° angles, together with a possible Arg204 bonding at 3.73 Å and 171.6°. Gallic acid forms a compact polar cluster dominated by Asp206, Asp340, and Arg344, while Asp297 H-bond also can be seen from the figure. Asp206 is observed at 3.54 Å and 111.7°, Asp340 at 3.70 Å and 135.5°, and Arg344 at 3.04–3.10 Å with 144.1–146.8° angles. Quercetin exhibits the broadest acidic network in this receptor, with Asp168, Glu230, Asp297, Asp340, Arg344, Val171, Asp206, and Trp83 around the ligand. In line with this, the geometry analysis shows three contacts to Asp168 at 3.21–3.40 Å and 118.0–130.2°, a Glu230 contact at 4.00 Å and 109.3°, an Asp297 contact at 3.32 Å and 119.2°, an Asp340 contact at 3.67 Å and 109.4°, two Arg344 contacts at 2.93–3.21 Å and 132.5–141.7°, and a Val171 contact at 3.99 Å and 129.2°. Chlorogenic acid is anchored by the Asp297/Gln35/Arg344 side of the pocket, with additional participation of Asp206, Asp340, His122. It can also be seen that there are Tyr82 and Leu173 hydrophobic contacts and nearby Trp83 aromatic interaction. H-bonds to Asp340 at 3.65 Å and 146.6°, His122 at 3.80 Å and 112.6°, Asp206 at 3.86 Å and 117.9°, Asp297 at 3.06 Å and 138.7°, Arg344 at 3.80 Å as a possible salt bridge, and Gln35 at 2.85 Å and 107.3°. Apigenin-7-*O*-glucoside adopts a more localized pose, with Tyr155, Asp297, His80, Gly167, and Tyr75. Tyr155 contact is at 3.28 Å/139.0°, an Asp297 contact is at 3.22 Å/146.2°, a His80 contact at 3.40 Å/116.4°, and a Gly167 contact at 3.61 Å/161.2°.

The 2QV4 interactions seem well-aligned with Asp197 and/or Asp300 active site residues, with additional Tyr62, Gln63, Thr163, Arg195, and neighboring residues contributing according to ligand scaffold. Gallic acid is centered on the Asp197/Asp300 region and additionally reaches Tyr62 and Arg195. Geometry records Tyr62 at 3.65 Å and 123.5° as a possible H-bond, Asp197 at 2.98 Å and 121.7°, Asp300 at 3.04 Å and 139.4°, Ala198 at 3.69 Å and 113.0°, and Arg195 at 3.13 Å with 128.5° angles. Quercetin has a well-developed network involving Tyr62, Asp197, Gln63, His101, and Arg195, with Trp59 and Glu233 also visible in the schematic; the corresponding numbers are 2.96 Å/105.0° for Tyr62, 2.88 Å/109.7° for Asp197, 3.89 Å/168.9° for Gln63, 4.03 Å/124.4° for His101, and 3.33–3.90 Å/134.8–156.5° for Arg195. Chlorogenic acid is anchored most clearly by Asp300, Lys200, and Glu233, while also weakly contacting Thr163 and possibly Tyr151; in addition, the figure places Trp59, Tyr62, Leu162, and Ala198 around the ligand as hydrophobic neighbors. Geometric analysis provides values to Thr163 at 3.04 Å and 103.4°, Lys200 at 3.05 Å and 118.0°, Asp300 at 2.87 Å and 157.6°, and Tyr151 at 4.01 Å and 104.3°. Ferulic acid shows a simpler arrangement focused on Asp300, Asp197, and Gln63. Leu165 interacts hydrophobically and Asp197 is also shown nearby; the geometric analysis numbers are 3.02 Å/123.8° for Asp300, 3.02 Å/105.0° for Thr163, and 2.98 Å/150.5° for Gln63, alongside a Leu165 hydrophobic contact at 3.60 Å. Apigenin-7-*O*-glucoside occupies the broadest 2QV4 footprint among the studied phytochemicals, with contacts to Trp59 at 3.39 Å/128.6°, Thr163 at 3.87 Å with 102.5–129.3° angles, Asp197 at 2.96 Å/136.7°, His305 at 3.29 Å/116.4°, Arg195 at 2.89–3.38 Å/124.9–138.1°, and Ala198 at 3.87 Å/103.6°.

In the 4GQR diagrams, the binding pattern again centers on the catalytic acidic region, but with stronger participation of Arg195, Gln63, and Tyr62/Trp59 interactions depending on chemical involved. Chlorogenic acid is stabilized by a number of H-bonds/possible polar contacts with Gln63 contact at 3.37 Å with 171.4° angles, Asp197 contacts at 2.90–3.14 Å with 107.8–118.8° angles, a Thr163 contact at 3.21 Å and 124.1°, and Arg195 contact at 3.11 Å with 130.1° angles; hydrophobic contacts are also present to Leu165 at 3.78–3.79 Å and Tyr62 at 3.78 Å. Ferulic acid retains the Tyr62/Trp59 side of the pocket and simultaneously engages Glu233, Asp197, Ala198, and Arg195 with the following numbers; to Tyr62 at 3.33 Å/117.5°, Glu233 at 3.45 Å/118.8°, Asp197 at 2.80 Å/108.4°, Ala198 at 3.92 Å/110.6°, and Arg195 at 3.08–3.93 Å with 130.8–174.3° angles, together with hydrophobic contacts to Trp59 at 3.52 Å and Tyr62 at 3.61 Å. In 4GQR, gallic acid showed the least connected pose, mainly involving His101 (3.23 Å/111.7°), Asp197 (3.92 Å/104.4°), and Asp300 (3.73 Å/119.8°). Quercetin shows a bit broader connectivity, with Trp59, Tyr62, Gln63, Asp197, Glu233, Ala198, and Arg195 around the ligand. Corresponding contacts to Trp59 at 3.66 Å/126.0°, Tyr62 at 3.35 Å/134.7°, Gln63 at 2.94–3.31 Å with 138.8–143.8° angles, Asp197 at 3.98 Å/125.2°, Glu233 at 3.19 Å/144.5°, Ala198 at 3.90 Å/115.0°, and Arg195 at 2.80 Å with 127° angles along with pi-stacking interactions with Tyr62 and Trp59. Apigenin-

7-*O*-glucoside extends further toward the Asp300/His305 side of the pocket, with geometric analysis showing contacts to Gln63 at 3.71 Å/105.9°, Asp197 at 3.69 Å/114.6°, Asp300 at 3.49–3.91 Å with 136.4–146.9° angles, His305 at 3.61 Å/126.1°, Thr163 at 3.48 Å/137.8°, and Ala307 at 3.00 Å/102.1°.

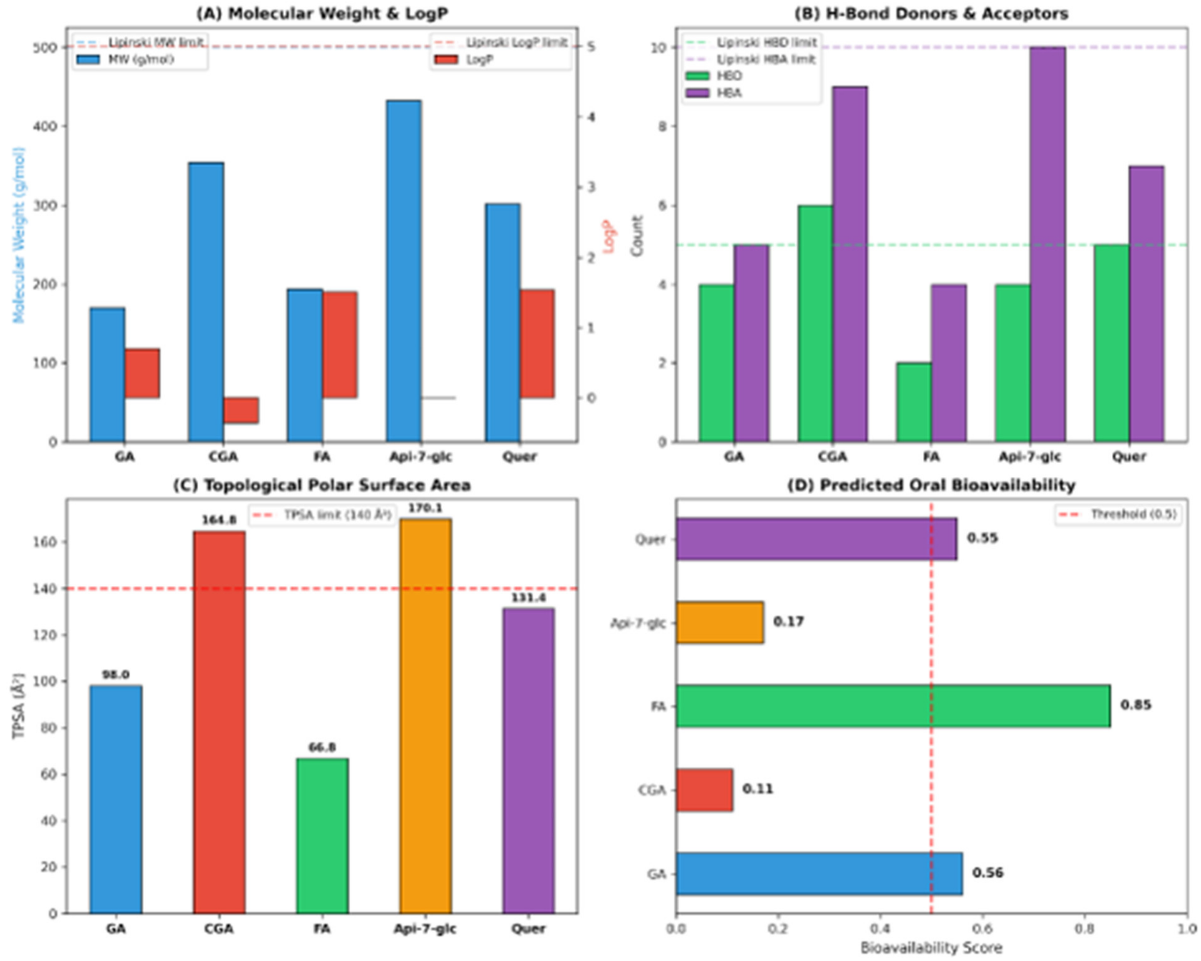

Figure S5. Physicochemical, pharmacokinetic (ADME) and drug-likeness properties of the identified phenolic markers, predicted with SwissADME: (A) molecular weight and LogP; (B) hydrogen-bond donors and acceptors; (C) topological polar surface area; (D) predicted oral bioavailability score. Toxicity endpoints were not predicted.

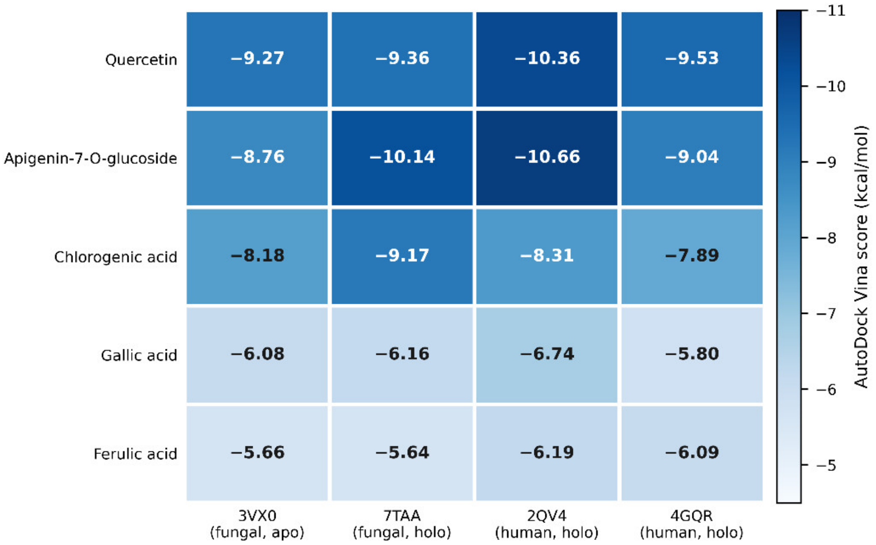

Figure S6. Molecular docking binding affinity heat map based on AutoDock Vina scores (kcal/mol) for the identified phenolic markers across 3VX0, 7TAA, 2QV4, and 4GQR.

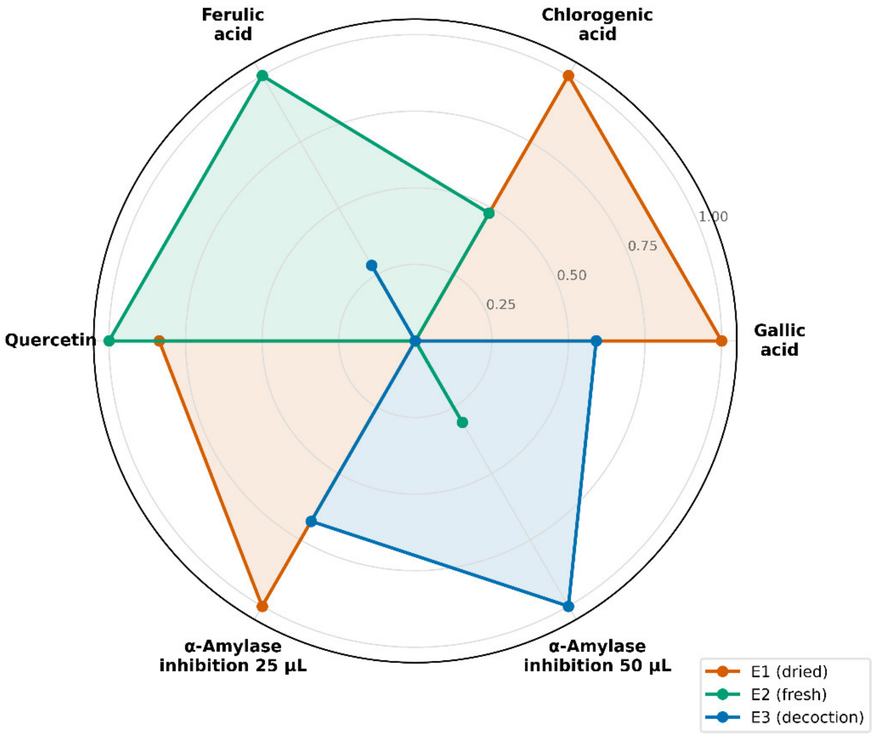

Figure S7. Comparative radar plot of normalized phenolic marker levels and α-amylase inhibition responses across E1, E2, and E3.

References

S1. Burley, S.K.; Bhikadiya, C.; Bi, C.; Bittrich, S.; Chen, L.; Crichlow, G.V.; Christie, C.H.; Dalenberg, K.; Di Costanzo, L.; Duarte, J.M.; et al. RCSB Protein Data Bank: Powerful new tools for exploring 3D structures of biological macromolecules for basic and applied research and education in fundamental biology, biomedicine, biotechnology, bioengineering and energy sciences. *Nucleic Acids Res.* 2021, *49*, D437–D451. doi:10.1093/nar/gkaa1038.

S2. Sugahara, M. Crystal structure of alpha-amylase from *Aspergillus oryzae*. RCSB Protein Data Bank, 2012, PDB ID 3VX0. doi:10.2210/pdb3VX0/pdb.
S3. Davies, G.J.; Brzozowski, A.M. Family 13 alpha amylase in complex with acarbose. RCSB Protein Data Bank, 1997, PDB ID 7TAA. doi:10.2210/pdb7TAA/pdb.
S4. Brzozowski, A.M.; Davies, G.J. Structure of the *Aspergillus oryzae* alpha-amylase complexed with the inhibitor acarbose at 2.0 Å resolution. *Biochemistry* 1997, *36*, 10837–10845. doi:10.1021/bi970539i.
S5. Williams, L.K.; Maurus, R.; Brayer, G.D. Human pancreatic alpha-amylase complexed with nitrite and acarbose. RCSB Protein Data Bank, 2007, PDB ID 2QV4. doi:10.2210/pdb2QV4/pdb.
S6. Maurus, R.; Begum, A.; Williams, L.K.; Fredriksen, J.R.; Zhang, R.; Withers, S.G.; Brayer, G.D. Alternative catalytic anions differentially modulate human α-amylase activity and specificity. *Biochemistry* 2008, *47*, 3332–3344. doi:10.1021/bi701652t.
S7. Williams, L.K.; Brayer, G.D. Human pancreatic alpha-amylase in complex with myricetin. RCSB Protein Data Bank, 2012, PDB ID 4GQR. doi:10.2210/pdb4GQR/pdb.
S8. Williams, L.K.; Li, C.; Withers, S.G.; Brayer, G.D. Order and disorder: Differential structural impacts of myricetin and ethyl caffeate on human amylase, an antidiabetic target. *J. Med. Chem.* 2012, *55*, 10177–10186. doi:10.1021/jm301273u.
S9. Jurrus, E.; Engel, D.; Star, K.; Monson, K.; Brandi, J.; Felberg, L.E.; Brookes, D.H.; Wilson, L.; Chen, J.; Liles, K.; et al. Improvements to the APBS biomolecular solvation software suite. *Protein Sci.* 2018, *27*, 112–128. doi:10.1002/pro.3280.
S10. Olsson, M.H.M.; Søndergaard, C.R.; Rostkowski, M.; Jensen, J.H. PROPKA3: Consistent treatment of internal and surface residues in empirical pK_a predictions. *J. Chem. Theory Comput.* 2011, *7*, 525–537. doi:10.1021/ct100578z.
S11. Kim, S.; Chen, J.; Cheng, T.; Gindulyte, A.; He, J.; He, S.; Li, Q.; Shoemaker, B.A.; Thiessen, P.A.; Yu, B.; et al. PubChem 2023 update. *Nucleic Acids Res.* 2023, *51*, D1373–D1380. doi:10.1093/nar/gkac956.
S12. Ropp, P.J.; Kaminsky, J.C.; Yablonski, S.; Durrant, J.D. Dimorphite-DL: An open-source program for enumerating the ionization states of drug-like small molecules. *J. Cheminform.* 2019, *11*, 14. doi:10.1186/s13321-019-0336-9.
S13. O'Boyle, N.M.; Banck, M.; James, C.A.; Morley, C.; Vandermeersch, T.; Hutchison, G.R. Open Babel: An open chemical toolbox. *J. Cheminform.* 2011, *3*, 33. doi:10.1186/1758-2946-3-33.
S14. Trott, O.; Olson, A.J. AutoDock Vina: Improving the speed and accuracy of docking with a new scoring function, efficient optimization, and multithreading. *J. Comput. Chem.* 2010, *31*, 455–461. doi:10.1002/jcc.21334.
S15. Eberhardt, J.; Santos-Martins, D.; Tillack, A.F.; Forli, S. AutoDock Vina 1.2.0: New docking methods, expanded force field, and Python bindings. *J. Chem. Inf. Model.* 2021, *61*, 3891–3898. doi:10.1021/acs.jcim.1c00203.
S16. McNutt, A.T.; Francoeur, P.; Aggarwal, R.; Masuda, T.; Meli, R.; Ragoza, M.; Sunseri, J.; Koes, D.R. GNINA 1.0: Molecular docking with deep learning. *J. Cheminform.* 2021, *13*, 43. doi:10.1186/s13321-021-00522-2.
S17. Li, H.; Leung, K.-S.; Wong, M.-H.; Ballester, P.J. Improving AutoDock Vina using random forest: The growing accuracy of binding affinity prediction by the effective exploitation of larger data sets. *Mol. Inform.* 2015, *34*, 115–126. doi:10.1002/minf.201400132.
S18. Wójcikowski, M.; Zielenkiewicz, P.; Siedlecki, P. Open Drug Discovery Toolkit (ODDT): A new open-source player in the drug discovery field. *J. Cheminform.* 2015, *7*, 26. doi:10.1186/s13321-015-0078-2.
S19. Buttenschoen, M.; Morris, G.M.; Deane, C.M. PoseBusters: AI-based docking methods fail to generate physically valid poses or generalise to novel sequences. *Chem. Sci.* 2024, *15*, 3130–3139. doi:10.1039/D3SC04185A.
S20. RDKit. RDKit: Open-Source Cheminformatics, version 2023.09.1, 2023. Available online: https://www.rdkit.org (accessed on 15 March 2026).
S21. Adasme, M.F.; Linnemann, K.L.; Bolz, S.N.; Kaiser, F.; Salentin, S.; Haupt, V.J.; Schroeder, M. PLIP 2021: Expanding the scope of the protein–ligand interaction profiler to DNA and RNA. *Nucleic Acids Res.* 2021, *49*, W530–W534. doi:10.1093/nar/gkab294.
S22. Meli, R.; Biggin, P.C. spyrmsd: Symmetry-corrected RMSD calculations in Python. *J. Cheminform.* 2020, *12*, 49. doi:10.1186/s13321-020-00455-2.
S23. Leung, S.; Bodkin, M.; von Delft, F.; Brennan, P.; Morris, G. SuCOS is better than RMSD for evaluating fragment elaboration and docking poses. *ChemRxiv* 2019, preprint. doi:10.26434/chemrxiv.8100203.v1.
S24. Pettersen, E.F.; Goddard, T.D.; Huang, C.C.; Meng, E.C.; Couch, G.S.; Croll, T.I.; Morris, J.H.; Ferrin, T.E. UCSF ChimeraX: Structure visualization for researchers, educators, and developers. *Protein Sci.* 2021, *30*, 70–82. doi:10.1002/pro.3943.

S25. Keskin, E.; Mısırlı, D.; Elmastaş, M. Phenolic content analysis and antioxidant activity of decoction and ethanolic extracts obtained from dried and fresh *Arum maculatum*. *J. Integr. Anatol. Med.* **2025**, *6*, 9–16. doi:10.53445/batd.1680521.
